# Combining morphological and genomic evidence to resolve species diversity and study speciation processes of the *Pallenopsis patagonica* (Pycnogonida) species complex

**DOI:** 10.1186/s12983-019-0316-y

**Published:** 2019-09-06

**Authors:** Jana S. Dömel, Till-Hendrik Macher, Lars Dietz, Sabrina Duncan, Christoph Mayer, Andrey Rozenberg, Katherine Wolcott, Florian Leese, Roland R. Melzer

**Affiliations:** 1 Aquatic Ecosystem Research, Faculty of Biology, University of Duisburg-Essen, Universitaetsstr. 5, 45141 Essen, Germany; 2 Zoological Research Museum Alexander Koenig, Statistical Phylogenetics and Phylogenomics, Adenauerallee 160, 53113 Bonn, Germany; 3 Bavarian State Collection of Zoology – SNSB, Muenchhausenstr. 21, 81247 Munich, Germany; 4 Faculty of Biology, Technion – Israel Institute of Technology, 3200003 Haifa, Israel; 5 Centre for Water and Environmental Research (ZWU), University of Duisburg-Essen, Universitaetsstr. 2, 45141 Essen, Germany; 6 Department Biologie II, LMU Munich, Großhaderner Str. 2, 82152 Planegg-Martinsried, Germany; 7 GeoBioCenter, LMU Munich, Richard-Wagner-Str. 10, 80333 Munich, Germany

**Keywords:** Sea spider, Marine benthos, Antarctica, Patagonia, Integrative taxonomy, Target hybrid enrichment, Cryptic species, Selection

## Abstract

**Background:**

*Pallenopsis patagonica* (Hoek, 1881) is a morphologically and genetically variable sea spider species whose taxonomic classification is challenging. Currently, it is considered as a species complex including several genetic lineages, many of which have not been formally described as species. Members of this species complex occur on the Patagonian and Antarctic continental shelves as well as around sub-Antarctic islands. These habitats have been strongly influenced by historical large-scale glaciations and previous studies suggested that communities were limited to very few refugia during glacial maxima. Therefore, allopatric speciation in these independent refugia is regarded as a common mechanism leading to high biodiversity of marine benthic taxa in the high-latitude Southern Hemisphere. However, other mechanisms such as ecological speciation have rarely been considered or tested. Therefore, we conducted an integrative morphological and genetic study on the *P. patagonica* species complex to i) resolve species diversity using a target hybrid enrichment approach to obtain multiple genomic markers, ii) find morphological characters and analyze morphometric measurements to distinguish species, and iii) investigate the speciation processes that led to multiple lineages within the species complex.

**Results:**

Phylogenomic results support most of the previously reported lineages within the *P. patagonica* species complex and morphological data show that several lineages are distinct species with diagnostic characters. Two lineages are proposed as new species, *P. aulaeturcarum* sp. nov. Dömel & Melzer, 2019 and *P. obstaculumsuperavit* sp. nov. Dömel, 2019, respectively. However, not all lineages could be distinguished morphologically and thus likely represent cryptic species that can only be identified with genetic tools. Further, morphometric data of 135 measurements showed a high amount of variability within and between species without clear support of adaptive divergence in sympatry.

**Conclusions:**

We generated an unprecedented molecular data set for members of the *P. patagonica* sea spider species complex with a target hybrid enrichment approach, which we combined with extensive morphological and morphometric analyses to investigate the taxonomy, phylogeny and biogeography of this group. The extensive data set enabled us to delineate species boundaries, on the basis of which we formally described two new species. No consistent evidence for positive selection was found, rendering speciation in allopatric glacial refugia as the most likely model of speciation.

**Electronic supplementary material:**

The online version of this article (10.1186/s12983-019-0316-y) contains supplementary material, which is available to authorized users.

## Background

The diversity of the marine benthos of the Southern Hemisphere has been influenced by large scale extension of grounded glaciers on the Patagonian and Antarctic continental shelves during repeated glacial cycles in the Plio- and Pleistocene [17, 29, 78]. Several studies suggested that benthic life was limited to few isolated refugia in which independent divergence and lineage sorting processes promoted today’s high species diversity in Southern Ocean and Patagonian shelf habitats [1, 15, 30, 34, 51]. Molecular taxonomic studies added evidence on the role of glacial impacts on species divergence by reporting many previously unrecognized species (often referred to as “cryptic species”) over the last few decades that often show non-overlapping, allopatric distribution ranges [1, 39, 51, 83].

One animal group with remarkable (cryptic) species diversity are sea spiders [20, 25, 26, 48, 57, 81]. Sea spiders, or pycnogonids, are a group of exclusively marine arthropods that are especially diverse in the Southern Ocean [6].

One prominent example for high species diversity is the *Pallenopsis patagonica* (Hoek, 1881) [42] sea spider species complex. *Pallenopsis patagonica* has a holobenthic life cycle and is reported to occur with a circumpolar distribution around sub-Antarctic islands and on the continental shelf of Antarctica as well as southern South America [62], i.e. in regions that were strongly impacted by glaciations during the last ice ages [58]. Since its first description by Hoek [42], several authors have commented on the high morphological variability of *P. patagonica* and suggested that it represents a species complex [32, 40, 55, 81]. However, species delineation within this complex is difficult and there is a long history of attempts to resolve this question by either splitting the species when describing new species often based on a small number of specimens (e.g. [40, 41, 61, 68]), or by lumping several species together declaring them synonymous (e.g. [13]). This culminated in two drastically different surveys by Pushkin [69] and Child [13]. While Pushkin [69] described more new species for the species complex, Child [13] refuted this and instead recognized only one, *P. patagonica*, to which he attributed a high variability. At the moment, four formerly described species are considered synonyms of *P. patagonica*: *P. glabra* (Möbius, 1902) [61], *P. hiemalis* (Hodgson, 1907) [40], *P. meridionalis* (Hodgson, 1915) [41] and *P. moebiusi* (Pushkin, 1975) [68, 7, 13]. Furthermore, there are more closely related species from the Southern Hemisphere whose relationship to or position within the species complex is unclear, e.g. *P. buphtalmus* (Pushkin, 1993) [69], *P. latefrontalis* (Pushkin, 1993) [69], *P. macneilli* (Clark, 1963) [14] and *P. notiosa* (Child, 1992) [12]. Hence, several studies have addressed this issue in recent years by adding genetic data. First, Weis et al. [81] reported that mostly sub-Antarctic specimens previously assigned to *P. patagonica* can be genetically divided into several groups based on mitochondrial cytochrome *c* oxidase subunit I (COI) data. Weis et al. [81] also reported high morphological variability within the species complex. Based on the genetic and morphological differences, a new species was described, named *P. yepayekae* (Weis, 2014) [81]. Further groups within the species complex were suggested based on molecular data reported by Harder et al. [37] for Antarctic *P. patagonica* specimens. The authors defined ten distinct clades (labelled A-J) using the mitochondrial COI marker [37]. To validate the proposed number of clades and to exclude mito-nuclear discordances, which can be found in other pycnogonids, e.g. *Colossendeis megalonyx* (Hoek, 1881) [42, 20], Dömel et al. [26] investigated the highly variable nuclear internal transcribed spacer (ITS) marker for previously studied clades. In contrast to *C. megalonyx*, most lineages of the *P. patagonica* species complex were supported by both markers (only a few recently diverged ones were not). Thus, no evidence for mito-nuclear discordance was found. This suggested that the distinct lineages represented species defined based on the biological species concept. With additional specimens studied by Dömel et al. [26], additional clades were identified. Altogether, 19 clades with mostly regional distribution patterns were proposed as independently evolving lineages under the specific name *patagonica* (labelled ANT A-N and SUB 1–5 in [26], according to their geographic occurrence).

So far, no diagnostic morphological characters are known to delineate clades and characterise new species within the *P. patagonica* species complex, which, however, would be critically important in order to assess the benthic diversity of the Southern Hemisphere and test hypotheses regarding the underlying evolutionary processes.

Many studies on benthic invertebrates, especially on benthic brooders that lack pelagic larval stages like sea spiders, have interpreted the fact that species typically showed allopatric distribution patterns as evidence for lineage sorting in independent ice-free refugia [1, 38, 39, 51, 74].

However, one study on the sea slug *Doris kerguelenensis* (Bergh, 1884) [8] that occurs in the Southern Ocean, as well as sub-Antarctic waters, suggested that interspecific competition for prey was involved in speciation [83]. Similarly, Rutschmann et al. [70] tested for adaptive speciation and radiation in notothenioid fish and found lineage-independent ecological differentiation into different niches probably as a result of positive selection. This provides evidence that consideration of genetic drift and independent lineage development in isolated refugia may not suffice to explain the enormous diversity in southern marine benthic habitats [16, 33]. In fact, Antarctic and sub-Antarctic waters bear such a diverse range of extreme and different habitats and display diverse biotic interactions that speciation due to ecological divergence should more explicitly be explored as a potential process for speciation. In order to test for evidence of selection, quantitative evidence for functionally relevant changes in the genome has to be provided. With the availability of new analytical techniques for morphology (e.g. micro-computed tomography; μCT) and genetics (Next Generation Sequencing (NGS), e.g. target hybrid enrichment; [27, 59]), it becomes possible to generate large integrative data sets. Target hybrid enrichment, i.e. a technique that captures specific genes with known homology across a taxonomic group using synthetic probes, offers an immense potential to test for genes under selection, especially in poorly studied organisms such as all Southern Hemisphere marine benthic invertebrate species. Hence, this method can also be used to further investigate the species diversity and to test competing hypotheses and compare neutral vs. non-neutral speciation hypotheses, i.e. lineage sorting in bottlenecked refugia vs. adaptive divergence. By combining genomic and morphometric data sets, greater morphological differences are expected especially for taxa living in sympatry in contrast to those living in allopatry due to potential niche specialisation in form of ecological character displacement [18, 19, 72].

Therefore, in this study we integrate all previous data on the *P. patagonica* species complex, combine them with genomic data obtained via target hybrid enrichment, analyses of morphological features using conventional observation methods and meristic data to study patterns of diversity and underlying evolutionary processes within the *P. patagonica* species complex. Specifically, we address the following questions:

Do genome-wide data add further information about previously unrecognised species diversity within the *P. patagonica* species complex?

Do we find morphological characters to distinguish the independently evolving lineages of the *P. patagonica* species complex and formally describe new species?

Do we find evidence for adaptive divergence at morphological or genetic level or do neutral evolutionary processes suffice to explain the observed species diversity?

## Results

The sample set included specimens of *Pallenopsis buphtalmus* (corresponding to mitochondrial clade ANT_M in [26]), *P. latefrontalis* (ANT_F), *P. notiosa* (SUB_3) and *P. yepayekae* (Pye.1) as well as of further potential species within *P. patagonica*, i.e. ANT_C, ANT_D, ANT_K, ANT_L, SUB_1, SUB_2, SUB_4 and SUB_5. We refer to this set of putative species as the *P. patagonica* species complex (also *P. patagonica* sensu lato in [26]), since using the key in Child [12] would (erroneously) assign all those species to the morphospecies *P. patagonica*.

### Genomic analyses

The obtained dataset consisted of 61 individuals of the *Pallenopsis patagonica* species complex. One individual of *P. pilosa* (Hoek, 1881) [42] genotyped by us and a previously published transcriptome assembly of *Anoplodactylus insignis* (Hoek, 1881) [42] [28] were added as outgroups in genetic analyses. When analyzing all *Pallenopsis* specimens on the nucleotide level, 821 out of 1607 targeted EOGs (Eukaryotic Orthologous Groups), which in our case are putative single-copy groups of orthologous genes, were recovered with a total alignment length of 474,954 bp. The data set used to infer a reliable root by including *A. insignis* was analysed on the amino acid level to reduce the branch length to the outgroup. This alignment included only EOGs for which a sequence of *A. insignis* was present and sites with a sequence coverage of at least 50%, which reduced the data set to 208 EOGs and 22,018 aa (corresponding to 66,054 bp). Furthermore, sequences that were outliers on the amino acid level were excluded. The models of evolution chosen by ModelFinder for the nucleotide data set were GTR + R2 for the first, TIM + R2 for the second and GTR + R4 for the third codon positions. For the amino acid alignment including *A. insignis*, JTT + F + R3 was chosen as the best fitting model.

Single-nucleotide polymorphism (SNP) calling for all *Pallenopsis* samples (i.e. including *P. pilosa*) resulted in 2527 SNPs from 168 EOGs. This data set was only used for construction of a phylogenetic tree.

**Fig. 1:**
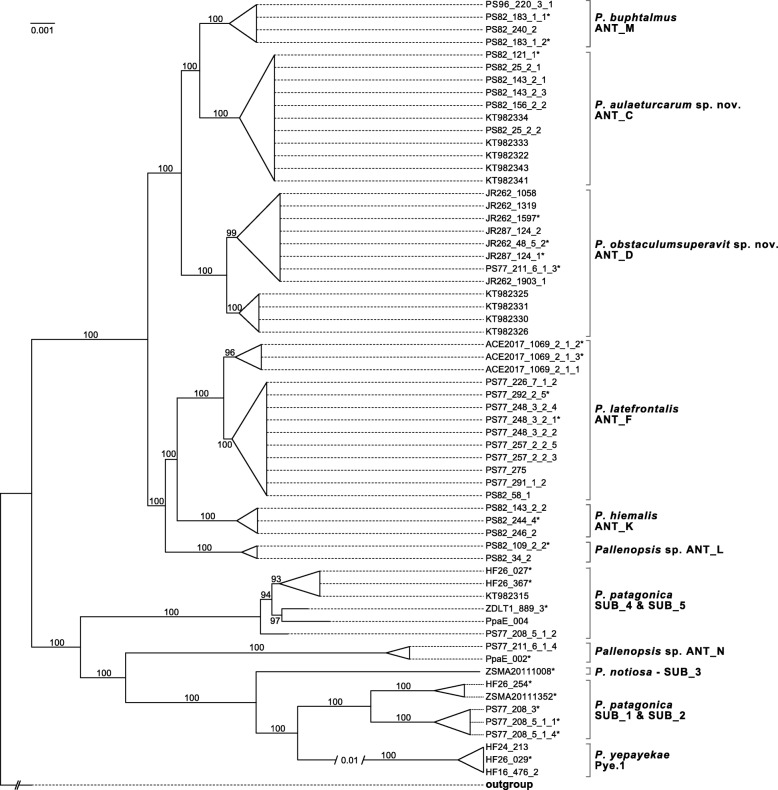
Phylogenetic tree of the *Pallenopsis patagonica* species complex. Maximum-Likelihood tree based on concatenated EOG sequences of all *Pallenopsis* samples. Asterisks (*) indicate samples that were also used in morphometric analyses. Bootstrap values are given next to the respective branches

Phylogenetic analyses of the amino acid data set revealed that the *P. patagonica* species complex represents a monophyletic group with *P. pilosa* and *A. insignis* representing a joint outgroup (Additional file 1). In particular *P. pilosa* was shown to be a sister group to the *P. patagonica* species complex, as assumed in previous studies [26, 81]. Further analyses were conducted with the nucleotide data set not including *A. insignis*. Separate phylogenetic analyses based on the EOG alignment (in the following referred to as the EOG data set) and the variant calling (in the following referred to as the SNP data set) including all *Pallenopsis* specimens resulted in phylograms with identical topologies (Fig. 1 and Additional file 2) but the EOG-based analysis had higher bootstrap support (bs) values and is discussed herein. Two major groups are discernible within the *P. patagonica* species complex, one including specimens assigned to all of the Antarctic clades (ANT) except ANT_N (from now on referred to as the “Antarctic supergroup”) and one including specimens from all Patagonian clades (SUB) plus ANT_N (from now on referred to as “Patagonian supergroup”). The “Antarctic supergroup” is comprised of two major lineages, ANT_C/D/M and ANT_F/K/L. More detailed divisions of those groups are in agreement with the clades delineated in Dömel et al. [26]. There is also a strong support for the geographical divide in ANT_D and *P. latefrontalis* (ANT_F) into specimens from the Antarctic shelf (both 100% bs) and sub-Antarctic islands (South Georgia with 99% bs, and Bouvet Island with 96% bs, respectively). Within the “Patagonian supergroup”, SUB_4 and SUB_5 together represent the basalmost group of the “Patagonian supergroup” with SUB_4 being paraphyletic with respect to SUB_5. Analogously, SUB_1 and SUB_2 appear not strictly monophyletic with respect to each other, since specimens from Burdwood Bank belonging to both clades group together. ANT_N is nested within the “Patagonian supergroup”, as are *P. notiosa* (SUB_3) and *P. yepayekae*.

**Fig. 2:**
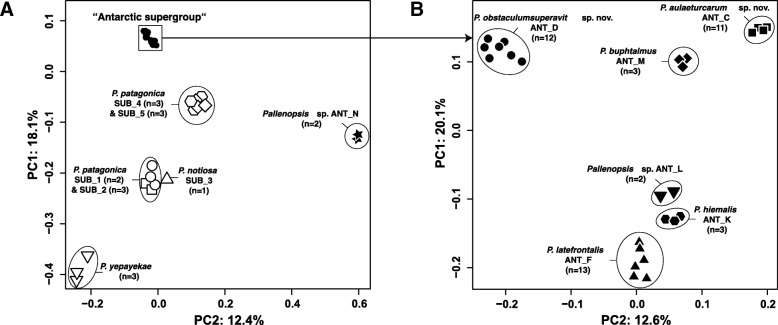
PCA from genomic data of the *Pallenopsis patagonica* species complex. PCA plots based on genomic data of **a**) all samples of the *Pallenopsis patagonica* species complex and **b**) samples of the “Antarctic supergroup”

For the principal component analyses (PCA) three SNP data sets were analysed. The first data set contained all specimens of the *P. patagonica* species complex and included 2543 SNPs from 175 EOGs. Furthermore, separate data sets for the “Patagonian supergroup” and the “Antarctic supergroup” yielded 2047 SNPs from 183 EOGs and 2487 SNPs from 216 EOGs, respectively. For the first SNP data set (*P. patagonica* species complex), 16 significant axes were found. There is a clear differentiation between five groups (Fig. 2a). All Antarctic clades cluster together, with the exception of ANT_N. The Patagonian clades are divided into four groups, SUB_1/2, *P. notiosa* (SUB_3), SUB_4/5 and *P. yepayekae* (Pye.1). Analyses of the data set divided into the two supergroups obtained no significant axes for the “Patagonian supergroup”. For the “Antarctic supergroup”, the first seven axes were significant and showed a differentiation into the clades previously proposed by Dömel et al. [26] (Fig. 2b).

**Fig. 3:**
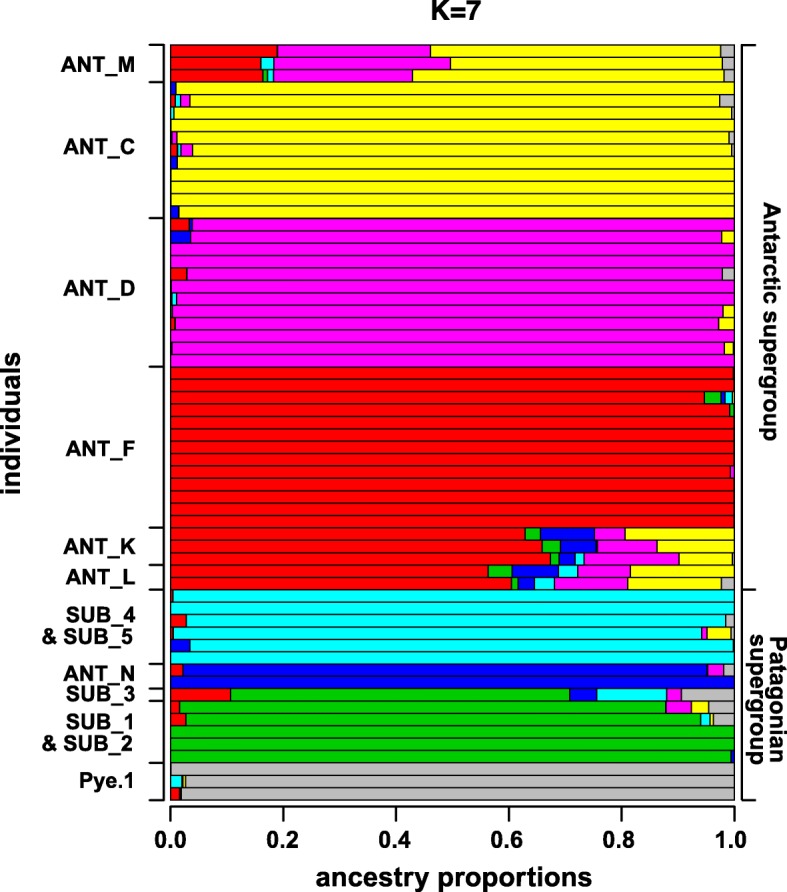
sNMF analyses of the *Pallenopsis patagonica* species complex. Graphical illustration of ancestry proportion estimates for all samples with K = 7. Estimated proportions of ancestry populations are illustrated by different colors. Each horizontal bar represents one specimen

For the clustering analyses, the cross entropy with the lowest median was chosen (Additional file 3). By this criterion, the best number of ancestral populations was seven (K = 7). The plot of the sparse nonnegative matrix factorization (sNMF) mostly supported the groupings obtained with the PCA. The differences were that ANT_K and ANT_L as well as SUB_1 and SUB_2 grouped together and showed similar proportions of the same ancestral populations (Fig. 3).

For selection tests, a sequence alignment including only positions that were present in at least 50% of the samples was used. This resulted in an alignment of 82,782 bp recovering 293 EOGs. Seventeen codons within 17 EOGs and 49 codons within 38 EOGs under selection using the Fast Unconstrained Bayesian AppRoximation (FUBAR) and the Mixed Effects Model of Evolution (MEME), respectively, were detected. Sixteen codons within 16 EOGs were shared between both methods. Furthermore, no branches under selection were detected, irrespective of the applied test (aBSREL or BUSTED; see Methods).

### Morphology

#### Morphometrics

Morphometric measurements were taken for 37 individuals (a table including all measurements is provided in Additional file 4) but due to damage during trawling, transport, storage or preceding genetic analysis, distal articles of appendices and hence data for those were often missing. After averaging measurements for bilateral characters, the amount of missing data was reduced by about three quarters. For further analyses, filtered data sets including 38 and 39 characters for the absolute and relative values, respectively, were used. PCA plots using all specimens did not show separation into clades but a trend for a division of sub-Antarctic and Antarctic samples (Additional file 5).

To avoid the problem of overfitting, character sets optimal for species separation in discriminant analysis (LDA) were searched for using a heuristic approach. Therefore, only clades with a minimum of three individuals were included resulting in a data set of seven clades and 29 specimens. Absolute as well as relative values expressed as proportion of the trunk length were used.

**Fig. 4:**
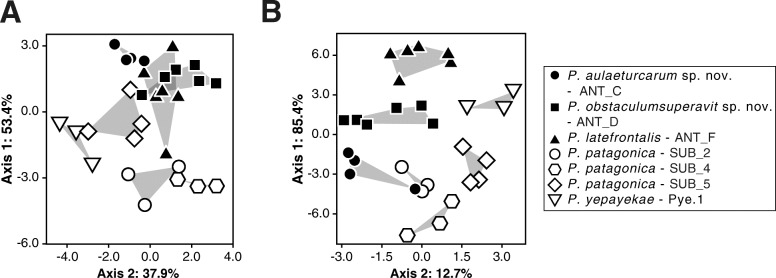
LDA of the *Pallenopsis patagonica* species complex. Ordination of the filtered morphometric data set using different combination of characters for **a**) absolute values (ocular tubercle H, ocular tubercle W, eye H, forehead H, cheliphore 1), and **b**) relative values (trunk W12, ocular tubercle H, palp, cheliphore 2, WL4 coxa2)

For both data sets (absolute and relative values) multiple iterations of character selection were performed and it was recorded how often a character was added to an LDA model in individual optimizations and what its contribution was (see Table 1). The LDA plots of both data sets based on the character combinations with best performance clearly separated all clades from each other, except for clade ANT_D and ANT_F when looking at the absolute values (Fig. 4). Furthermore, analysis of cross-validation confusion matrices confirmed that these results were not dominated by overfitting artefacts, with the correctness rate being higher for the relative values (0.83) than for the absolute values (0.76) (Table 2). Here, ANT_F and SUB_5 had many misassignments (absolute data set). Analogously, PCAs for both data sets showed that the clades ANT_D and ANT_F could not be separated from each other for the data set including absolute values (see matrices of all PCs in the Additional file 6).

**Table 1:** Results of morphometric analyses of the *Pallenopsis patagonica* species complex for both data sets (absolute and relative values). Contributions to correctness rate (CR), characters combination for the best LDA performance and *p*-values for significant differences between geographic regions and sexes are listed

Character	Absolute					Relative				
	Times the character contributed to an LDA model during character selection^a^	Increase in correctness rate (mean ± SD)^b^	Character combination for best LDA performance	Differences between geographic regions *	Differences between sexes *	Times the character contributed to an LDA model during character selection^a^	Increase in correctness rate (mean ± SD)^b^	Character combination for best LDA performance	Differences between geographic regions *	Differences between sexes *
abdomen W	34 (2.4%)	0.05 ± 0.03		< 0.001	–	19 (1.23%)	0.09 ± 0.04		–	–
abdomen L	27 (1.9%)	0.21 ± 0.15		< 0.001	–	2 (0.13%)	0.09 ± 0.01		–	–
eye H	115 (8.11%)	0.22 ± 0.11	x	< 0.001	–	15 (0.97%)	0.1 ± 0.06		–	–
eyes distance	6 (0.42%)	0.06 ± 0.03		–	–	22 (1.43%)	0.08 ± 0.03		–	–
ocular tubercle W	15 (1.06%)	0.05 ± 0.03	x	0.004	–	24 (1.56%)	0.08 ± 0.05		–	–
ocular tubercle H	147 (10.37%)	0.1 ± 0.07	x	–	–	102 (6.63%)	0.12 ± 0.05	x	0.005	0.012
ceph. segment	36 (2.54%)	0.09 ± 0.08		< 0.001	–	24 (1.56%)	0.11 ± 0.06		0.012	–
cheliphore 1	136 (9.59%)	0.2 ± 0.13	x	< 0.001	–	2 (0.13%)	0.11 ± 0.01		0.048	–
cheliphore 2	NA	NA	NA	NA	NA	5 (0.32%)	0.09 ± 0.03	x	–	–
cheliphore 3	5 (0.35%)	0.05 ± 0.02		< 0.001	–	1 (0.06%)	0.1		0.027	–
cheliphore 4	8 (0.56%)	0.06 ± 0.03		0.001	–	23 (1.49%)	0.09 ± 0.04		–	–
palp	41 (2.89%)	0.11 ± 0.1		0.001	–	198 (12.87%)	0.16 ± 0.08	x	–	–
proboscis thick2tip	136 (9.59%)	0.11 ± 0.06		–	–	83 (5.39%)	0.15 ± 0.08		–	–
proboscis basis	23 (1.62%)	0.11 ± 0.07		< 0.001	0.027	6 (0.39%)	0.06 ± 0.05		–	–
proboscis thickest	29 (2.05%)	0.17 ± 0.14		< 0.001	–	7 (0.45%)	0.07 ± 0.05		0.001	–
proboscis L	2 (0.14%)	0.02 ± 0.01		< 0.001	–	13 (0.84%)	0.06 ± 0.03		< 0.001	–
trunk W1	90 (6.35%)	0.11 ± 0.07		< 0.001	–	41 (2.66%)	0.11 ± 0.05		0.004	0.022
trunk W12	30 (2.12%)	0.09 ± 0.06		0.048	–	168 (10.92%)	0.17 ± 0.09	x	< 0.001	–
trunk W2	8 (0.56%)	0.07 ± 0.05		0.001	–	18 (1.17%)	0.07 ± 0.05		–	–
trunk W23	55 (3.88%)	0.08 ± 0.05		–	–	81 (5.26%)	0.12 ± 0.06		< 0.001	–
trunk W3	98 (6.91%)	0.1 ± 0.07		< 0.001	–	61 (3.96%)	0.1 ± 0.06		0.034	–
trunk W34	15 (1.06%)	0.06 ± 0.03		–	–	73 (4.74%)	0.11 ± 0.05		< 0.001	0.042
trunk W4	5 (0.35%)	0.03 ± 0		< 0.001	–	23 (1.49%)	0.15 ± 0.09		–	0.037
trunk H	1 (0.07%)	0.03		–	–	20 (1.3%)	0.1 ± 0.04		–	–
trunk L	48 (3.39%)	0.11 ± 0.11		< 0.001	–	NA	NA		NA	NA
forehead H	69 (4.87%)	0.07 ± 0.04	x	0.029	–	45 (2.92%)	0.09 ± 0.04		–	–
WL1 coxa1	17 (1.2%)	0.16 ± 0.08		< 0.001	–	45 (2.92%)	0.1 ± 0.05		–	–
WL1 coxa2	27 (1.9%)	0.1 ± 0.09		0.001	–	60 (3.9%)	0.12 ± 0.06		0.012	0.034
WL1 coxa3	4 (0.28%)	0.07 ± 0.03		0.002	–	3 (0.19%)	0.09 ± 0.05		–	–
WL1 femur	7 (0.49%)	0.04 ± 0.02		< 0.001	0.009	NA	NA		NA	NA
WL2 coxa1	NA	NA		NA	NA	15 (0.97%)	0.06 ± 0.03		–	–
WL2 coxa2	20 (1.41%)	0.17 ± 0.12		0.001	–	66 (4.29%)	0.17 ± 0.11		0.041	0.016
WL2 coxa3	NA	NA		NA	NA	4 (0.26%)	0.08 ± 0.04		–	–
WL3 coxa1	7 (0.49%)	0.11 ± 0.05		0.001	–	21 (1.36%)	0.09 ± 0.04		–	0.023
WL3 coxa3	12 (0.85%)	0.2 ± 0.1		< 0.001	0.008	19 (1.23%)	0.08 ± 0.05		< 0.001	–
WL4 coxa1	5 (0.35%)	0.18 ± 0.13		< 0.001	–	1 (0.06%)	0.07		–	–
WL4 coxa2	59 (4.16%)	0.19 ± 0.14		< 0.001	–	160 (10.4%)	0.16 ± 0.1	x	–	0.042
WL4 coxa3	2 (0.14%)	0.07 ± 0		< 0.001	0.038	3 (0.19%)	0.08 ± 0.04		–	–
WL4 propodus	8 (0.56%)	0.14 ± 0.16		< 0.001	–	22 (1.43%)	0.09 ± 0.04		–	–
WL4 tarsus	11 (0.78%)	0.1 ± 0.06		< 0.001	0.025	2 (0.13%)	0.08 ± 0.06		–	–
WL4 tibia2	60 (4.23%)	0.2 ± 0.13		< 0.001	–	42 (2.73%)	0.11 ± 0.06		–	–

* *p*-values are only listed for analyses that showed significant differences. ^a^Number of times the charcter was added and its addition led to a positive increase in cross-validation correctness rate of individual LDA models during repeated character selections (% of total in parentheses). See how the repetitions were organized in Materials and Methods. ^b^Average increase in cross-validation correctness rate after addition of the character to an LDA model had a positive effect in the character selections

**Table 2:** Cross-validation confusion matrices for morphometric data set of the *Pallenopsis patagonica* species complex using absolute and relative values

	absolute (correctness rate: 0.76)							relative (correctness rate: 0.83)						
	ANT_C	ANT_D	ANT_F	Pye.1	SUB_2	SUB_4	SUB_5	ANT_C	ANT_D	ANT_F	Pye.1	SUB_2	SUB_4	SUB_5
ANT_C	4	0	0	0	0	0	0	3	0	0	0	1	0	0
ANT_D	1	5	0	0	0	0	0	1	5	0	0	0	0	0
ANT_F	1	0	3	0	1	0	1	0	0	6	0	0	0	0
Pye.1	0	0	0	3	0	0	0	0	0	0	3	0	0	0
SUB_2	0	0	0	0	3	0	0	1	0	0	0	2	0	0
SUB_4	0	0	0	0	0	3	0	0	0	0	0	0	2	1
SUB_5	1	0	1	1	0	0	1	0	0	0	1	0	0	3

Significant differences of characters between clades were found for neither of the two data sets after Bonferroni correction. However, 33 and 14 significant differences between specimens from the different geographic regions (SUB and ANT) for absolute and relative value, respectively, were found (Table 1). In all cases, the characters of the Antarctic samples were larger than of the Patagonian ones. As for analyzed specimens, males were more frequent in sub-Antarctic (75%) and females preponderated in Antarctic clades (65%), characters were also tested for significant differences between sexes. There were five and eight significant differences for absolute and relative values, respectively, of which five characters for each data set also showed significant differences between geographic regions (see Table 1).

#### Morphological characters

**Fig. 5:**
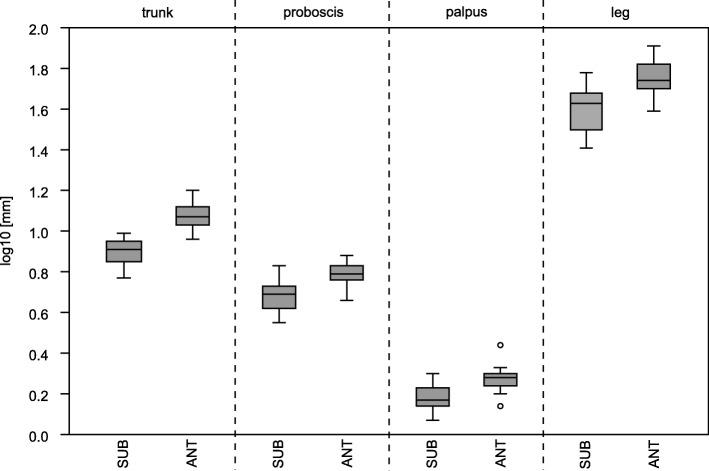
Boxplot showing size differences in morphological structures of the *Pallenopsis patagonica* species complex. All comparisons show that characters of samples from ANT (Antarctica) are significantly larger than from SUB (Patagonian) (log10 of absolute values used; *p* = 0.0000005, *p* = 0.00008, *p* = 0.00042 and *p* = 0.00012, respectively)

Using the morphological key for *Pallenopsis* [82] from [13], all specimens analysed were assigned to *P. patagonica*. However, we observed consistent morphological features for several groups. Specimens that occur south of the Antarctic Polar Front are larger in body size and have longer legs in comparison to those from the Patagonian clades. Also, the distance between the lateral processes is longer for the Antarctic specimens. Furthermore, the rudimentary palp is larger for Antarctic individuals (Fig. 5).

**Fig. 6:**
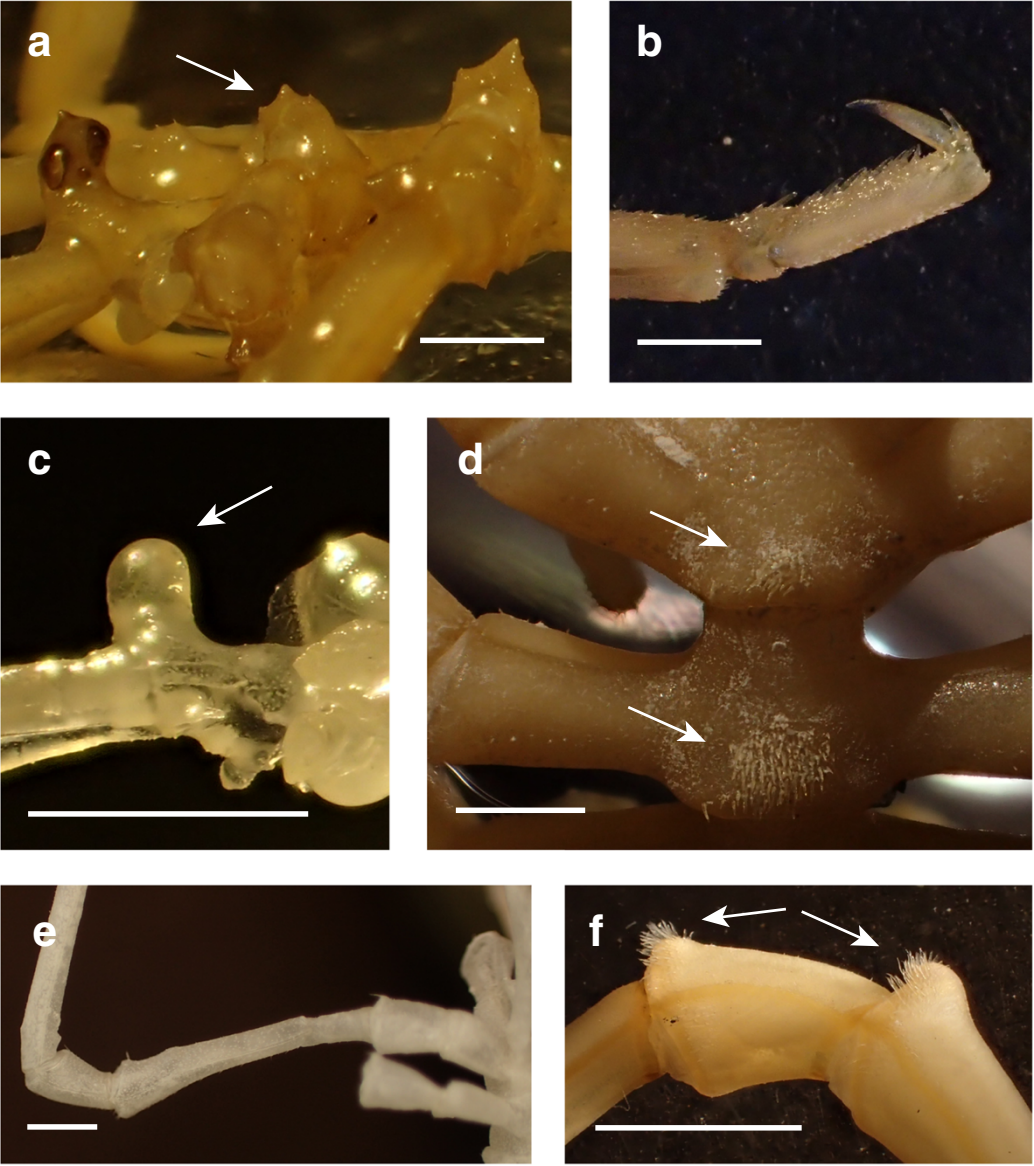
Prominent morphological characters of various lineages of the *Pallenopsis patagonica* complex. **a**, dorso-distally located crowning (see arrow) of lateral processes in PS82_143_2_2 (*P. hiemalis*; ANT_K). **b**, straight propodus of PS82_143_2_2 (*P. hiemalis*; ANT_K). **c**, rounded ocular tubercle of ZSM-A20111008 (*P. notiosa*; SUB_3). **d**, setae patches (see arrows) on dorsal-posterior margin of three trunk segments of JR262_1058 (*P. aulaeturcarum*; ANT_D). **e**, coxae of ZSM-A20111008 (*P. notiosa*; SUB_3). **f**, detailed view of second and third coxa with bifurcated setae on distal margins (see arrows) of PS77_211_6_1_4 (*Pallenopsis* sp. ANT_N). Scale bars = 1.5 mm

Specimens from Patagonian clades showed great variation and almost no suitable morphological characters for clade assignments. Only *P. notiosa* (SUB_3) can be distinguished from the others due to its rounded (rather than a pointed or slightly pointed) ocular tubercle and a very long second coxa, which exceeds the combined lengths of the first and third coxae (Fig. 6c,e).

Specimens from Antarctica can morphologically be divided into two groups which can be distinguished by the setae patches on the dorso-posterior margin of the trunk segments (Fig. 6d), that vary in size for specimens of ANT_C/D/M but are absent in those of ANT_F/K/L and ANT_N. Two Antarctic clades were identified as already described species, namely *P. buphtalmus* (ANT_M) and *P. latefrontalis* (ANT_L). *Pallenopsis buphtalmus* (ANT_M) can be distinguished from the other Antarctic species due to relatively short accessory claws. For *P. latefrontalis* (ANT_L) the second coxa is characteristically shorter than the combined lengths of the first and third coxae. A straight rather than a curved propodus is distinctive of ANT_K (Fig. 6b). Also, the lateral processes in this clade display a dorso-distally located crowning that differs from the frequently occurring but much smaller thickenings (Fig. 6a). Those characters were also described for *P. hiemalis* by Hodgson [40] and Pushkin [68, 69] by their slim segmented body, cylindrical proboscis, rudimentary palps, ten-articled ovigera in males, and slender legs with one main and two auxiliary claws [82].

### *Pallenopsis aulaeturcarum* sp. nov. Dömel & Melzer urn:lsid:zoobank.org:act:72E41F8B-0A6F-4A5B-815A-1C2CAB65AFA5 Figures 7 a-g, 9 a-e

#### Type material

Holotype: PS82_156_2_1 (ZSM-A20160629), female, Weddell Sea, − 75.507 (S), − 27.486 (W), January 2014, depth: 281.5 m.

Paratypes: PS82_121_1 (ZSM-A20160626), female, Weddell Sea, − 76.966 (S), − 32.945 (W), January 2014, depth: 265.2 m. First leg pair and ovigera loose in the jar, proboscis of this individual was used for further analyses with the scanning electron microscope (SEM); PS82_156_2_2 (ZSM-A20160630), female, Weddell Sea, − 75.507 (S), − 27.486 (W), January 2014, depth: 281.5 m; PS82_223_1 (ZSM-A20160730), male, Weddell Sea, − 75.522 (S), − 28.973 (W), February 2014, depth: 462 m, both ovigera damaged, cement gland tube used for sex determination; PS82_174_3 (ZSM-A20160637), male, Weddell Sea, − 74.491 (S), − 30.977 (W), February 2014, depth: 529.7 m, left oviger detached, no morphometric measurements available for this individual.

The type series is deposited in the Bavarian State Collection of Zoology, in the department Arthropoda varia.

##### Distribution

Weddell Sea, from eastern tip of the Antarctic Peninsula (− 63.686, − 56.859) to eastern Weddell Sea (− 70.940, − 10.489), and Bouvet Island (− 54.425, 3.524).

##### Diagnosis

Setae on posterior margin of trunk segments. More rows on ventral side (about three) than on dorsal side (one row). Abdomen oriented upwards.

##### Description (female)

**Fig. 7:**
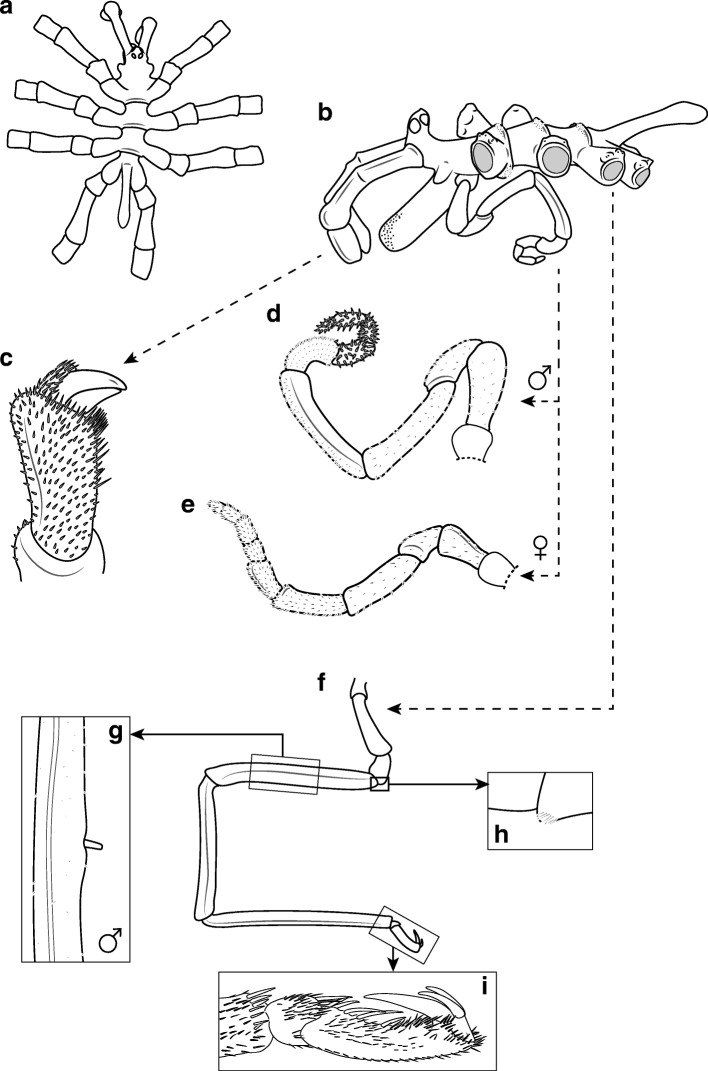
*Pallenopsis aulaeturcarum* sp. nov. Dömel & Melzer (clade ANT_C). (**a**), dorsal view. (**b**), lateral view of male. (**c**), cheliphore. (**d**), male oviger. (**e**), female oviger. (**f**), walking leg with enlargement of cement gland tube (**g**), setae on third coxa (**h**), and propodus with claw and auxiliary claws (**i**)

Size moderate, leg span less than 65 mm. Trunk with distinct segment borders, ridges strongly expressed (Fig. 7a,b). Ridges on dorsal side smooth with few setae. Ventral surface covered with 2–3 rows of small clearly apparent spinules. Lateral processes separated by about the size of their diameter, U-shaped (Figs. 7a, 9a). Distal margins of all processes display fringe of small spinules. On dorsal side, these spinules are located on slight thickenings (Fig. 7b). Ocular tubercle situated on anterior end of cephalic segment. Top of ocular tubercle slightly bent backwards and pointed. Eyes prominent and pigmented, anterior eyes larger than posterior eyes. Proboscis sub-cylindrical, equally thick throughout and slightly directed downwards (Fig. 9a,b). It is about half the length of the trunk. Abdomen long, extending from the trunk oriented upwards and covered with few spinules (Figs. 7b, 9a). Cheliphores with two-articled scape, first article longer than second article (Fig. 7b). Ultimate cheliphore article (movable finger) equipped with setose pad. Moveable digit slightly longer than fixed digit, its tip curved. Inner margins straight and joined when closed. Setae pad has a triangular shape of which the whole length is attached to chela. Single-articled, laterally placed palp represents the rudimentary state typical for the genus (Fig. 7b). It takes the form of an elongated bulb that is twice as long as wide. Female oviger composed of ten articles (Fig. 7e). Proximal articles broaden slightly towards the distal part of each article. Second article equal in length to the third article. Fourth oviger article more swollen and the longest of all. From fourth article onwards, article length decreases. Oviger articles are setose, with all setae pointing distally. Legs with several short setae (Fig. 7f,g). First and third coxa sub-equal. Second coxa about twice the length of third coxa (Fig. 7a,f). Assemblage of short setae on ventral side of second and third coxa (Fig. 7h, 9d). Setae without bifurcation. Femur and first tibia about equal in size. Second tibia slightly longer than other leg articles. Tarsus is short and armed with one big spine on the ventral side near its distal part and a couple of smaller lateral spines. Propodus slightly curved, with three to four heel spines that differ insignificantly in length, but the distal spine is the largest (Fig. 7i, Fig. 9e). The remaining sole is covered with many shorter spines. Claw dorsally curved, its inner margin straight, its tip curved. Two auxiliary claws about half the length of main claw. Sexual pores on all second coxae on ventrodistal surface. In contrast to the male, the female lacks cement gland tubes (see below).

##### Measurements (holotype in mm)

Length of trunk (anterior margin of first trunk segment to distal margin of fourth lateral processes), 9.80; trunk width (across first lateral processes), 3.98; proboscis length, 4.27; abdomen length, 3.91; third right leg, coxa 1, 1.45; coxa 2, 4.68; coxa 3, 2.78; femur, 13.74; tibia 1, 11.66; tibia 2, 15.08; tarsus, 0.32; propodus, 2.46; claw, 1.72; auxiliary claws, 0.63.

Different segments were measured in natural posture.

##### Male

The general habitus and size of the male are similar to those of the female. Differences are in the sexual characters: oviger ten-articled (as is typical for the genus), but longer than in the female (Fig. 7d). Second articles, nearly twice the length of third article. Fourth and fifth articles the longest and equal in size. Distal articles more setose than proximal articles, with setae pointing in various directions. Long cylindrical cement gland tube is located in the center of the ventral side of the femur in a small recess on top of a little swelling (Figs. 7g, 9c). It is about a third of the diameter of the femur and points away from the podomere’s surface in a nearly right angle. Sexual pores on ventral side of second coxae of third and fourth pair of legs.

##### Etymology

The specific name *aulaeturcarum* stands for “the yard (aula) of the Turks (turcae)” and is dedicated to the eponymous pub in Munich called “Türkenhof” that was frequently visited to discuss the complex and very variable morphology of *Pallenopsis*. The good atmosphere and drinks definitely improved the spirit and inspired the authors.

##### Remarks

This species belongs to the *Pallenopsis patagonica* s.l. species complex as defined in [26] and also analysed in [37]. In the previous studies, this species was defined as clade ANT_C or C, respectively.

There are no unique characters present for this new species which can be used to separate it from most other species of the genus, but the combination of its several diagnostic characters (shape of cheliphore pad, distances of lateral processes, presence of setae on ventral and dorsal side of trunk, as well as absence of long setae on legs and thickenings on lateral processes) makes it possible to distinguish it from the others.

### *Pallenopsis obstaculumsuperavit* sp. nov. Dömel urn:lsid:zoobank.org:act:69F7ADB8-26BB-4183-A178-67EEBABAE8BE Figures 8 a-g, 9 f-j

#### Type material

Holotype: JR262_1058 (ZSM-A20160708), female, South Georgia, − 55.144, − 36.245, 195.21 m, November/December 2011, missing legs: 3rd and 4th right side, 4th left side; one loose leg in the jar.

Paratypes: JR262_48_5_2 (ZSM-A20160713), female, South Georgia, − 54.284, − 36.083, 124.08 m, November/December 2011; JR287_124_1 (ZSM-A20160691), male; South Georgia, − 53.764, − 36.681, 151 m, May 2013; JR287_152 (ZSM-A20160694), female, South Georgia, − 53.758, − 36.690, 145 m, May 2013, Proboscis of this individual was used for further analyses with the SEM; JR262_1597_2 (ZSM-A20160710), male, South Georgia, − 54.396, − 37.384, 174.98 m, November/December 2011; PS77_211_6_1_3 (ZSM-A20160696), female, Shag Rocks, − 53.402, − 42.668, 290.2 m, February 2011.

The type series is deposited in the Bavarian State Collection of Zoology, in the department Arthropoda varia.

##### Distribution

Southern Ocean, from sub-Antarctic islands (South Georgia and Shag Rocks; − 53.597, − 41.214) as well as the Antarctic continental shelf (west and east of the tip of the Antarctic Peninsula; − 63.389, − 60.120).

##### Diagnosis

Setae patches of half the width of lateral processes on first trunk segment and with size of width of whole lateral process for second and third trunk segment. Abdomen pointing downwards.

##### Description (female)

**Fig. 8:**
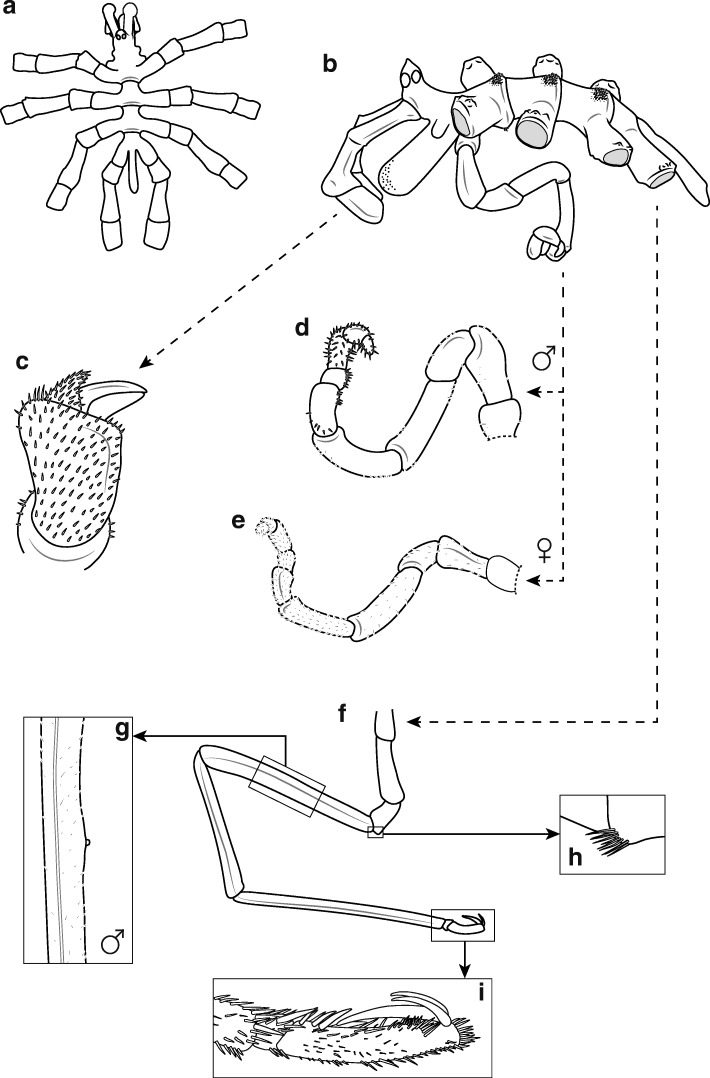
*Pallenopsis obstaculumsuperavit* sp. nov. Dömel (clade ANT_D). (**a**), dorsal view. (**b**), lateral view of male. (**c**), cheliphore. (**d**), male oviger. (**e**), female oviger. (**f**), walking leg with enlargement of cement gland tube (**g**), setae on third coxa (**h**), and propodus with claw and auxiliary claws (**i**)

**Fig. 9:**
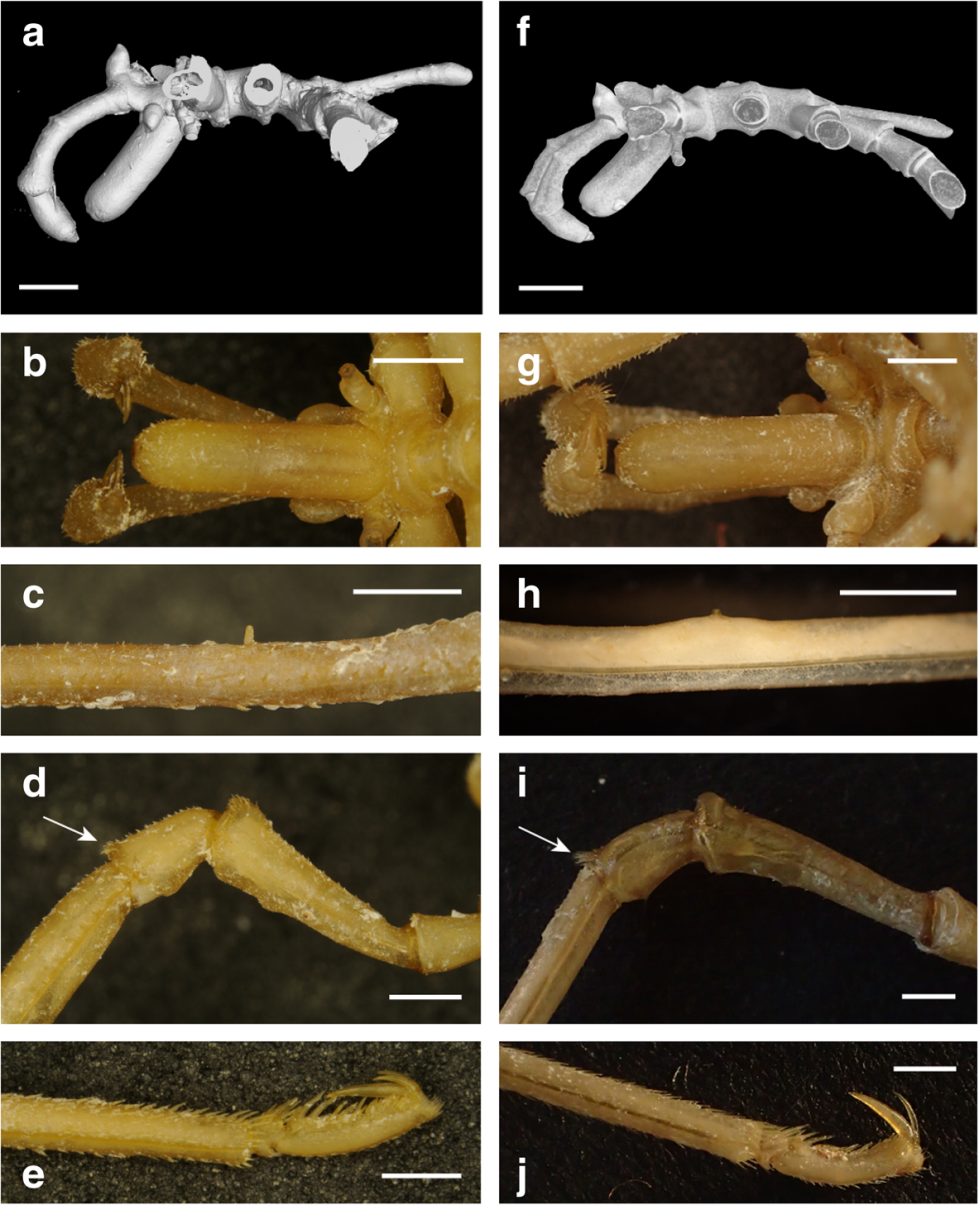
Images of *Pallenopsis aulaeturcarum* sp. nov. Dömel & Melzer (clade ANT_C) (**a**-**e**) and *Pallenopsis obstaculumsuperavit* sp. nov. Dömel (clade ANT_D) (**f**-**j**). **a**, **f**, micro-computed tomography (μCT) of specimens in lateral view; scale bar = 2.5 mm. **b**, **g**, ventral view of proboscis; scale bar = 1.5 mm. **c**, **h**, detail view of cement gland tube on femur (male); scale bar = 1.5 mm. **d**, **i**, detailed view of coxae with setae on posterior margin of the third coxa (see arrow); scale bar = 1.5 mm. **e**, **j**, propodus with claw and accessory claws; scale bar = 1.5 mm. **a**, PS82_121_1; **b**, **d**, **e**, PS82_156_2_1; **c**, PS82_185_1; **f**, JR287_152; **g**-**j**, JR287_124_3

Size moderate, leg span less than 85 mm. Trunk with distinct segment borders, ridges strongly expressed (Fig. 8a,b). Ridges on dorsal side setae-rich with a setae patch of half the width of lateral processes on first segment and with size of width of whole lateral process for second and third trunk segment. Ventral surface covered with few setae. Lateral processes separated by about the size of their diameter, U-shaped (Figs. 8a, 9f). Distal margins of all processes display fringe of small spinules. On dorsal side, these spinules are located on slight thickenings (Fig. 8b). Ocular tubercle situated on anterior end of cephalic segment. Top of ocular tubercle slightly bent backwards and pointed. Eyes prominent and pigmented, anterior eyes larger than posterior eyes. Proboscis sub-cylindrical, equally thick throughout and slightly directed downwards (Fig. 9f,g). It is about half the size of the trunk. Abdomen long, extending ventrally from the thorax and covered with few spinules (Figs. 8a, 9f). Cheliphores with two-articled scape, first article longer than second article (Fig. 8c). Ultimate cheliphore article (movable finger) equipped with setose pad. Moveable digit slightly longer than fixed digit, its tip curved. Inner margins straight and joined when closed. Setae pad has a triangular shape of which half the length is attached to chela whereas other half protrudes. Single-articled, laterally placed palp represents the rudimentary state typical for the genus (Fig. 8b). It takes the form of an elongated bulb that is twice as long as wide. Female oviger composed of ten articles (Fig. 8e). Proximal articles broaden slightly towards the distal part of each article. Second article longer, nearly twice the size of third article. Fourth oviger article more swollen and the longest of all. From fourth article onwards, article length decreases. Oviger articles are setose, with all setae pointing distally. Legs with several short setae (Fig. 8f,g). First and third coxa sub-equal. Second coxa about twice the length of third coxa. Assemblage of conspicuous setae on ventral side of second and third coxa, brush-like (Figs. 8h, 9i). Setae without bifurcation. Femur and first tibia about equal in size. Second tibia longest leg article. Tarsus is short and armed with one big spine on the ventral side nearer its distal part and a couple of smaller lateral spines. Propodus slightly curved, with three to four heel spines that differ insignificantly in length, but the distal spine is the largest (Figs. 8i, 9j). The remaining sole is covered with many shorter spines. Claw dorsally curved, its inner margin straight, its tip curved. Two auxiliary claws about one-half the length of main claw. Sexual pores on all second coxae on ventrodistal surface. In contrast to the male, the female lacks cement gland tubes (see below).

##### Measurements (holotype in mm)

Length of trunk (anterior margin of first trunk segment to distal margin of fourth lateral processes), 14.33; trunk width (across first lateral processes), 7.40; proboscis length, 6.35; abdomen length, 6.36; third right leg, coxa 1, 2.53; coxa 2, 7.59; coxa 3, 3.2; femur, 19.76; tibia 1, 15.91; tibia 2, 24.50; tarsus, 0.81; propodus, 4.43; claw, 2.46; auxiliary claws, 1.49.

Different segments were measured in natural posture.

##### Male

The general habitus and size of the male is similar to the female. Differences are in the sexual characters: oviger also ten-articled, typical for genus, but longer than female (Fig. 8d). Second articles longer, nearly twice the length of third article. Fourth and fifth articles the longest and equal in size. Distal articles more setose than proximal articles, with setae pointing in various directions. Small cylindrical cement gland tube is located in the center of the ventral side of the femur on top of a little swelling (Figs. 8g, 9h). It is as high as its diameter and points away from the podomere’s surface in a nearly right angle. Sexual pores on ventral side of second coxae of third and fourth pair of legs.

##### Etymology

The specific name *obstaculumsuperavit* stands for “the one that overcame (superare) the obstacle (obstaculum)”. *Pallenopsis obstaculumsuperavit* has been reported from the Antarctic continental shelf and South Georgia, which are separated by deep sea representing a barrier for the dispersal of many brooding invertebrates.

##### Remarks

This species belongs to the complex *Pallenopsis patagonica* s.l. defined in [26] and also analysed in [37]. In the previous studies, this species was defined as clade ANT_D or D, respectively.

### Combining morphological and genetic data

**Fig. 10:**
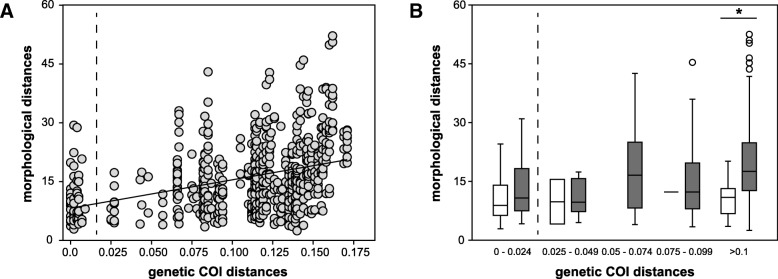
Morphological against genetic distances. Morphological distances plotted against uncorrected genetic COI distances **a**) for each individual with regression line (r = 0.36, *p* <  0.0001) and **b**) for genetic ranges differentiated into sympatric (white) and allopatric (grey) samples of the *Pallenopsis patagonica* species complex. Dashed line separates intraspecific (left) and interspecific (right) genetic distances

There is a significant positive correlation of greater morphological distances with larger genetic distances for both genetic distances calculated based on COI (r = 0.36, *p* <  0.0001; Fig. 10a) and EOG sequences (r = 0.51, p <  0.0001; see figure provided in the Additional file 7). There is only a small difference between both correlations and SUB_2 has high intraspecific genetic distances between specimens from Burdwood Bank and the Falkland Islands or the Patagonian shelf (Additional file 7). When dividing the genetic COI distances, which are available for all morphologically analysed individuals, into ranges (< 2.5% = intraspecific; > 2.5% = interspecific), the morphological distances are always higher for specimens that occur in allopatry than for those in sympatry. However, there is no significant difference between the genetic COI distance ranges, except for genetic distances above 10% (Fig. 10b).

## Discussion

### Do genome-wide data add further information about previously unrecognised species diversity within the *P. patagonica* species complex?

We successfully used the target hybrid enrichment method, with baits designed for a different genus [22], to obtain an unprecedented data set to resolve the taxonomy and phylogeny of the *Pallenopsis patagonica* species complex. The genomic data enabled us to obtain better resolved and stronger supported branches in the phylogenetic tree in comparison to the mitochondrial tree published in [26]. In general, the topologies of the trees were similar except for the placement of the root, which was placed on the branch leading to clade *Pallenopsis* sp. ANT_N in the mitochondrial tree. Genomic data revealed that all clades from the Patagonian shelf, including SUB_4 and SUB_5 that were found to be paraphyletic with respect to the Antarctic clades in the mitochondrial tree, grouped together in the “Patagonian supergroup”. In addition, *Pallenopsis* sp. ANT_N had a well-defined position within the “Patagonian supergroup” instead of being a sister taxon to all other species and clades of the *P. patagonica* species complex as in the previous analysis.

Most of the previously defined mitochondrial clades were well-separated in the multi-gene analyses with the exception of the two pairs of sister clades SUB_1/2 and SUB_4/5 (see table provided in Additional file 8 for an overview). It should be mentioned that the separation of these two pairs of clades was already put into question by the analysis of the ITS sequences in Dömel et al. [26]. There it was shown that specimens from Burdwood Bank (including one single specimen each from SUB_1 and SUB_2) grouped together, but had relatively large genetic distances. This disagreement with the mitochondrial clade assignment might be due to a mito-nuclear discordance, which has also been reported for the sea spider species complex *Colossendeis megalonyx* [20]. Although they were well-defined lineages in the phylogenetic tree, PCA and sNMF plots grouped both clades together. As morphological analyses also showed that there were no recognisable characters to distinguish SUB_1 and SUB_2, they should best be treated as one species. The differentiation between clades SUB_4 and SUB_5 was not supported by ITS data [26]. However, as no mito-nuclear discordance was found this could have been due to different mutation rates of the markers. The phylogenetic tree based on target hybrid enrichment revealed that SUB_4 is paraphyletic with respect to SUB_5, which may lead to the conclusion that this group originated on the Falkland Islands and subsequently migrated to the Patagonian shelf. Morphological data did not uncover characters to distinguish the two clades from each other and therefore support the hypothesis that they should still be considered as one species with geographical separation, as proposed by Dömel et al. [26]. Further intraspecific geographic separations were found for *P. obstaculumsuperavit* sp. nov. (ANT_D) and *P. latefrontalis* (ANT_F). For *P. obstaculumsuperavit* sp. nov. (ANT_D) a geographical differentiation has already been assumed between specimens from South Georgia and the Antarctic shelf based on the mitochondrial data set, but samples of *P. latefrontalis* (ANT_F) from Bouvet had not been analysed before. Geographic differentiation between populations from the Antarctic continental shelf and sub-Antarctic islands is known for other sea spiders [2, 25] as well as further benthic invertebrates [54, 79].

Principal component and phylogenetic tree analyses agreed with each other for all other predefined clades. But the cluster analysis showed similar proportions of ancestral populations for the closely related mitochondrial clades ANT_K (*P. hiemalis*, see below) and *Pallenopsis* sp. ANT_L together. However, the two clades were well separated in the phylogenetic tree and morphological analyses revealed several distinct characters between ANT_K and *Pallenopsis* sp. ANT_L. Therefore, we suggest that these clades represent two distinct species. Most likely, the relatively recent divergence of those two species in combination with a small sample size each (*n* = 3) represented an issue for the cluster analysis. Also, *P. notiosa* (SUB_3) clustered together with SUB_1/2 and again, this might be due to the small sample size especially of *P. notiosa* (SUB_3; *n* = 1).

The results of our analysis also allow to discuss questions on the biogeographic history of the *P. patagonica* species complex. Unlike previous studies based on few genes ([81, 37, 26]), our data clearly show a basal split between a Patagonian and an Antarctic group, whose distributions overlap only in South Georgia. As only little is known about the phylogeny of *Pallenopsis* as a whole and as we do not know exactly which species are the closest relatives of the *P. patagonica* species complex, we cannot assess whether the complex originated within Antarctica or not. However, the Antarctic supergroup shows a pattern of a relatively rapid radiation as opposed to the Patagonian supergroup which demonstrates earlier divergences. This pattern might be due to a rapid radiation after colonisation of the Antarctic, therefore supporting a non-Antarctic origin of the complex.

### Do we find morphological characters to distinguish the independently evolving lineages of the *P. patagonica* species complex and formally describe new species?

Using the key for *Pallenopsis* [82] from [13], we would characterize all specimens analysed as *P. patagonica*. This key, however, only includes nine out of 18 Antarctic and sub-Antarctic species [62]. The key given by Pushkin [69] for ten *Pallenopsis* of the Southern Ocean is misleading and would assign none of the analyzed specimens to *P. patagonica*. A recent attempt to update the identification key for Antarctic and sub-Antarctic *Pallenopsis* including all species was performed by Cano-Sánchez and López-González [9]. Still, not all specimens can be assigned correctly to species level. An example is *P. patagonica* (SUB_1/2/4/5), for which the lateral processes do not touch each other (but see [81]).

Morphometric analyses aiming at separating clades were challenging because of limited sample size. In addition, little is known regarding allometric growth in *Pallenopsis* and regression analysis was not possible for the same reason of not having sufficient numbers of individuals of both sexes for each clade [56]. Nevertheless, the simpler approach of taking relative lengths of morphological structures coupled with character selection for discriminant analysis showed that the species can be satisfactorily separated using a small number of characters with the relative values having better performance in species discrimination. The leg span best represents the actual body size of a sea spider and would have been the preferred reference length but analyses revealed cases of re-grown legs in the data set. Hence, relative values were expressed as proportions relative to the trunk lengths.

Diagnostic characters for at least nine species within the *P. patagonica* species complex, of which five have already been described, were found (see table provided in Additional file 8 for an overview). Additionally, Weis et al. [81] stated that *P. macneilli* a species found in Australian waters and hence was not included in this study, was also part of the *P. patagonica* species complex based on COI data. In general, the morphological distinction between genetic clades is clearer for the Antarctic ones. Weis et al. [81] already found out that the “Antarctic supergroup” consist of two described species, *P. buphtalmus* and *P. latefrontalis*. Furthermore, Weis et al. [81] mentioned that one specimen (PpaE002) stood out due to its horizontally positioned abdomen, in comparison to the common upwards oriented abdomen seen in most specimens. The above-mentioned individual has been genetically identified as *P. obstaculumsuperavit* sp. nov. (ANT_D). In fact, the position of the abdomen is a diagnostic character for this newly described species. The individual mentioned in Weis et al. [81] was reinvestigated and it can be confirmed that the horizontal position of the abdomen described before is actually downwards oriented, too.

*P. aulaeturcarum* sp. nov. (ANT_C) shares many morphological characters with other clades of the “Antarctic supergroup”, e.g. spinules on dorsal and ventral surface of the trunk, ratio of claw to accessory claw and propodus, and length of second coxa in relation to the sum of the first and third coxa. It should be stressed that the morphological differentiation would not have been recognised without the knowledge of the genetic background information thus highlighting once again the benefits of an integrative approach.

Cano-Sánchez and López-González [9] recently described two new species from Victoria Land (Ross Sea), *P. gracilis* Cano-Sánchez & López-González, 2019 [9] and *P. rotunda* Cano-Sánchez & López-González, 2019 [9]. Both can be distinguished from *P. obstaculumsuperavit* sp. nov. (ANT_D) by their upwards oriented abomina. Characters disagreeing with *P. aulaeturcarum* sp. nov. (ANT_C) are the lateral processes that are closer together, even touching, in *P. rotunda* and the forward pointing ocular tubercle of *P. gracialis*.

Specimens from the “Patagonian superclade” were morphologically very similar. In fact, SUB_1/2 and SUB_4/5 look alike and cannot be distinguished morphologically. If we were to consider the morphological result only, we would probably assign those clades to a single species. Strangely enough, within the phylogenetic tree *P. notiosa* (SUB_3), a well-defined species and *Pallenopsis* sp. ANT_N are placed between SUB_1/2 and SUB_4/5. Hence, SUB_1/2 and SUB_4/5 can be considered as cryptic but not sister species, a phenomenon that has also been observed, e.g. in nematodes [77].

Only two individuals from Shag Rocks (south of the Antarctic Polar Front) were available for *Pallenopsis* sp. ANT_N and therefore it was designated as an Antarctic clade by Dömel et al. [26]. Also morphologically, the two individuals were very similar to the Antarctic species (i.e. the distance between the lateral processes is about aslong as their diameter and the palps are longer than their diameter). However, phylogenetically, these specimens fell outside the Antarctic radiation and belonged to the “Patagonian supergroup”. Furthermore, during more detailed examination of these specimens, bifurcated setae, which are supposed to represent complex structures [52] with potential for species-specific features and (even if not as prominent) correspond to the character of the Patagonian species *P. yepayekae*, were detected on the second and third coxa. In fact, Weis et al. [81] described these setae as a unique character of specimens from the Chilean clade (i.e. *P. yepayekae*), and hence a character that can be used to distinguish it from specimens from the Antarctic region or the Falkland Islands. There were two species that were of particular interest, because they partly matched the characteristics of *Pallenopsis* sp. ANT_N: *P. tumidula* [55] and *P. candidoi* [60]. Both seemed to exhibit the short setae on the ventral side of the second and third coxa. The latter occurs from South Georgia to South Brazil and hence has a geographical overlap with *Pallenopsis* sp. ANT_N. *Pallenopsis candidoi* can be distinguished from *P. patagonica* s.s. and *P. yepayekae* by the eight-articled oviger in females, and by the auxiliary claws being clearly longer than half the lengthof the main claw [81]. The two individuals included in this study were males and no prediction can be made regarding the female ovigera, but the auxiliary claw isapproximately half the length of the main claw, rather than longer. *Pallenopsis* *tumidula* was characterised and drawn by Stock [75] with so-called ‘Fiederdornen’ (German for pinnated spine) on the ventral-distal side of the second and third coxa. He mentioned that this feature made *P.* *tumidula* clearly distinguishable from *P.* *patagonica*. Confusingly, in the original description of 1923, Loman neither mentioned short setae on the coxa nor depicted them in his drawings. Also, the originaldescription states that the lateral processes are separated by about half their diameter, which is smaller than those displayed by the studied specimens. However, due tothe small sample size, we refrain from designating this clade as a new species.

#### Reinstallment of P. hiemalis

Specimens assigned to clade ANT_K differed from the others in having a straight rather than curved propodus. Also, the lateral processes had a dorso-distally located crowning of up to three pointy tubercles that differs from the frequently occurring but much smaller thickenings. Those characters have also been described for *P. hiemalis* by Hodgson [40] and Pushkin [68, 69]. However, this species has been synonymised with *P. patagonica* by Child [13]. Cano-Sánchez and López-González [9] already suggested that *P. hiemalis* is a valid species, however this statement was made without any morphological reinvestigation. There are indeed characters in the original description of *P. hiemalis* that do not fit *P. patagonica* s.s. but are characteristic for Antarctic specimens of the species complex (e.g. “[…] lateral processes rather widely separated” and “Palps, a rather long stump”). Parts of the description that militate against ANT_K specimens being *P. hiemalis* concern the size of the second coxa, i.e. “[…] second [coxa] is fully twice as long as the other two together” [40]. This, however, is an uncommon ratio for *Pallenopsis* and also does not match the description of *P. patagonica* s.s. Hence, this might be a mistake due to a combination of the following phrasings: i) “[…] second [coxa] is twice as long as first or third coxa” and ii) “[...] second [coxa] is fully as long as the other two together”. The descriptions of *P. hiemalis* by Hodgson [40] and Pushkin [68] differ in their described characters, too. An example of this discordance is the description of a very prominent character of specimens from ANT_K which display three distinct tubercles on the dorsal-distal side of the lateral processes. Those were described as “tricipital tubercles” in Pushkin [68] but a single “stout tubercle of no great elevation” was described by Hodgson [40]. As Hodgson’s description is based on a single specimen, the missing character might be explained by a variation of attributes due to developmental stages. However, the few measurements given in Hodgson [40] indicate that the individual was full-grown. We herein propose to reinstall *P. hiemalis* [40] as autonomous species and refer to the species description in Pushkin [68]. *Pallenopsis hiemalis* belongs to *Pallenopsis patagonica* s.l. defined in [26] and also analyzed in [37]. In the previous studies, this species was referred to as clade ANT_K or K.

#### *Which species is* P. patagonica *s.s*.?

The original description of *P. patagonica* [42] agrees with the morphology of the specimens from clades SUB_4 (Falklands) and SUB_5 (Patagonia). Previous analyses revealed that those two clades can be distinguished with the mitochondrial COI but not with the nuclear ITS marker [26]. Further morphometric and morphological analyses detected no distinguishable characters and also the multi-marker analyses revealed that SUB_4 and SUB_5 can be considered as one species. Specimens of SUB_1/2 are very similar to those of SUB_4/5 and as the location of the type material of *P. patagonica* s.s. cannot be defined because the original description records specimens from three different locations in Patagonia (46°53′S 75°11′W, 50°10′S 74°42′W, and 52°20′S 68°0′W) where both species (SUB_1/2 and SUB_4/5) occur, it is difficult to decide which one represents *P. patagonica* s.s. A correct assignment of the species name *P. patagonica* to a genetic clade would therefore necessitate a genetic re-examination of the type series, which may not be obtainable from such old material.

#### Polar gigantism

The phenomenon that Antarctic specimens are unusually large is commonly known as polar gigantism [10]. The morphometric analyses revealed that all specimens within the Antarctic Polar Front are significantly larger than the Patagonian ones. This could have been biased by the fact that males dominated in sub-Antarctic and females dominated in Antarctic specimens. Size differences between male and female with the latter being the larger ones have been reported for many species [4], however dimorphism did not seem to influence our results.

Morphometric analyses alone would probably have led to incorrect conclusions regarding the phylogenetic position as one would have probably assumed that *Pallenopsis* sp. ANT_N is more closely related to the “Antarctic superclade”. However, looking at *Pallenopsis* sp. ANT_N in more detail, we detected bifurcated setae on the second and third coxa, which (even if not as prominent) is very similar to the character of the Patagonian species *P. yepayekae*. This can be seen as evidence for at least two independent events of polar gigantism within the genus *Pallenopsis*.

### Do we find evidence for adaptive divergence at morphological or genetic levels or do neutral evolutionary processes suffice to explain the observed species diversity?

Target hybrid enrichment can be used to specifically target coding regions and hence is a useful technique to test a large number of genes for selection [43]. For the *P. patagonica* species complex, only a few genes were found to be under selection. In addition, no branch under selection was detected and delineation due to selection pressure on any of the detected genes can be excluded. However, the bait set used here was not tailored to *P. patagonica* but was based on a transcriptome of the Southern Ocean sea spider *Colossendeis megalonyx*. Whereas for *C. megalonyx* all bait regions were recovered [22], for the *P. patagonica* species complex on average only 30% (max. of 35%) of all bait regions were successfully enriched. Most likely those loci represent well-conserved genes that show relatively little variation across families or genera of sea spiders and recently evolved genes that could have been of further relevance were not analyzed within this study.

Furthermore, there is no clear evidence for selection when analyzing morphological and genetic data together. In the case of sympatric speciation and adaptation to different ecological niches, one would expect high morphological differences also for recently diverged species, i.e. genetic distances just above 2.5%, and especially when they occur in sympatry (ecological character displacement). This does not appear to be the case in the *P. patagonica* species complex, because regardless of whether the species occur in sympatry or not, morphological distances were similarly high throughout the range of genetic distances. Only at very high genetic distances, for specimens living in allopatry morphological distances were significantly higher than for specimens living in sympatry. At the same time, specimens from the same area tended to be more similar to each other among species, which may be explained by their similar adaptations to the same environment.

Among the characters which were found to contribute to species separation we find some with potential ecological significance (like the absolute and relative length of the proboscis and first cheliphore article), which might indicate the existence of differences in food preferences between the species. The proboscis is known to have a diverse range of shapes and sizes among sea spiders indicating differences in feeding strategies [21, 80], but also the cheliphores can be relevant features as they are used to capture or cut the prey [4]. Yet, these characters are accompanied by other ones with supposedly little or no role in ecological differentiation. We might thus tentatively hypothesize that minor ecological differences between the species do exist, but they reflect local adaptation or even non-selective variation, since no character displacement in sympatry is observed. Especially species that occur in sympatry were expected to form different ecological niches. As the Antarctic continental shelf is relatively uniform in terms of geological structures and large regions that have been influenced by grounded ice shelfs or even iceberg ploughing are plain and dominated by gravel, food sources seem to be a major cause for specialisation. As this does not seem to be the case in the *P. patagonica* species complex, this might indicate that there is no competition for food. Jones [44] found a similar case were four species of the *Jaera albifrons* group (Crustacea; Isopoda) displayed identical mouthparts although they occurred in sympatry and concluded that food was not an isolating factor. It does not appear to be the case that the scarce morphological characters that differentiate the species of the complex, like position of the abdomen, distances between the lateral processes or shape of the setae patch on the cheliphores are of significant biological relevance and hence could be subject to selection.

## Conclusion

Combining genome-wide molecular sequence data with extensive morphological and morphometric analyses, we generated an unprecedented data set for members of the *P. patagonica* sea spider species complex. We established a well-resolved phylogeny based on target hybrid enrichment data and delineated species boundaries within the taxonomically difficult group which led to the reinstallment of *P. hiemalis* as well as the description of two new species, namely *P. aulaeturcarum* and *P. obstaculumsuperavit*. Contrary to previous studies, our results supported the division of the species complex into an Antarctic and a Patagonian group. Concerning speciation processes, our data supports the hypothesis of speciation in independent glacial refugia, as we found no consistent evidence for adaptive divergence. The latter aspect, however, can only be answered conclusively when more specimens from the different lineages and areas as well as more genomic loci become available.

## Methods

### Material

**Fig. 11:**
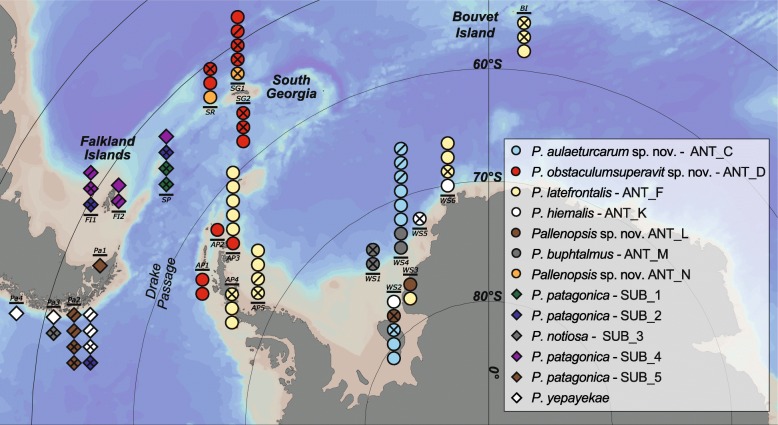
Specimen map. Sampling sites of Antarctic, sub-Antarctic and Patagonian specimens of the *Pallenopsis patagonica* species complex and their assignment to species or mitochondrial clades. Each symbol below or above the line and locality ID represents one specimen. Different clades are represented by different symbols/colors. Analysis methods are indicated for each individual within a symbol (slash: morphological analyses only; no indication: genetic analyses only; cross: genetic and morphological analyses)

A subset of specimens already included in Dömel et al. [26] was studied including individuals from the Antarctic continental shelf and the shelf of sub-Antarctic islands, the Falkland Islands and Patagonia (Fig. 11) (for further details of sampling and storage see [26]). Up to three individuals per species or clade were analyzed. For morphological measurements, 37 specimens were used. For genetic analyses, more samples of three lineages (ANT_C, ANT_D, *P. latefrontalis* (ANT_F)) with Antarctic distribution ranges were included and additional samples of *P. latefrontalis* (ANT_F) from Bouvet Island were added to improve the geographical coverage. Hence, the final genetic dataset consisted of 62 individuals of the *P. patagonica* species complex and a single individual of *P. pilosa* [42] as an outgroup (Table 3).

**Table 3:** List of specimens for the *Pallenopsis patagonica* species complex and outgroup used for target hybrid enrichment and morphometric measurements in this study

Species	Clade	ID	Location	Lat	Lon	Depth [m]	ZSM-Voucher Number	COI - GenBank/ BOLD Number	ITS- GenBank/ BOLD Number	Hybrid enrichment	Morphometric analyses
*Pallenopsis aulaeturcarum* sp. nov.	ANT_C	KT982322	AP3	−64.035	−56.728	220		KT982322	KY272414	x	
*P. aulaeturcarum* sp. nov.	ANT_C	KT982333	AP3	−63.686	−56.859	400		KT982333	KY272415	x	
*P. aulaeturcarum* sp. nov.	ANT_C	KT982334	AP3	−63.686	−56.859	400		KT982334	KY272416	x	
*P. aulaeturcarum* sp. nov.	ANT_C	KT982341	AP3	−63.754	−55.684	334		KT982341	KY272417	x	
*P. aulaeturcarum* sp. nov.	ANT_C	KT982343	AP3	−63.754	−55.684	334		KT982343	KY272418	x	
*P. aulaeturcarum* sp. nov.	ANT_C	PS82_25_2_1	WS4	−74.705	−29.900	406.2	ZSM-A20160635	KY272314	KY272404	x	
*P. aulaeturcarum* sp. nov.	ANT_C	PS82_25_2_2	WS4	−74.705	−29.900	406.2	ZSM-A20160636	KY272316	KY272406	x	
*P. aulaeturcarum* sp. nov.	ANT_C	PS82_121_1	WS2	−76.966	−32.945	265.2	ZSM-A20160626	KY272315	KY272412	x	x
*P. aulaeturcarum* sp. nov.	ANT_C	PS82_143_2_1	WS2	−76.967	−32.866	293.7	ZSM-A20160623	KY272311	KY272419	x	
*P. aulaeturcarum* sp. nov.	ANT_C	PS82_143_2_3	WS2	−76.967	−32.866	293.7	ZSM-A20160625	KY272319	KY272405	x	
*P. aulaeturcarum* sp. nov.	ANT_C	PS82_156_2_1	WS4	−75.507	−27.486	281.5	ZSM-A20160629	KY272313	KY272407		x
*P. aulaeturcarum* sp. nov.	ANT_C	PS82_156_2_2	WS4	−75.507	−27.486	281.5	ZSM-A20160630	KY272309	KY272413	x	
*P. aulaeturcarum* sp. nov.	ANT_C	PS82_156_2_3	WS4	−75.507	−27.486	281.5	ZSM-A20160631	KY272310	KY272410		x
*P. aulaeturcarum* sp. nov.	ANT_C	PS82_223_1	WS4	−75.522	−28.973	462	ZSM-A20160730	KY272308	KY272408		x
*P. obstaculumsuperavit* sp. nov.	ANT_D.1	KT982325	AP3	−63.576	−54.629	227		KT982325	KY272396	x	
*P. obstaculumsuperavit* sp. nov.	ANT_D.1	KT982326	AP2	−62.442	−55.459	245		KT982326		x	
*P. obstaculumsuperavit* sp. nov.	ANT_D.1	KT982330	AP1	−63.389	−60.120	310		KT982330		x	
*P. obstaculumsuperavit* sp. nov.	ANT_D.1	KT982331	AP1	−63.389	−60.120	310		KT982331		x	
*P. obstaculumsuperavit* sp. nov.	ANT_D.2	JR262_1058	SG2	−55.144	−36.245	195.21	ZSM-A20160708	KY272301		x	x
*P. obstaculumsuperavit* sp. nov.	ANT_D.2	JR262_1319	SG2	−55.002	−37.272	148.81	ZSM-A20160709	KY272302		x	
*P. obstaculumsuperavit* sp. nov.	ANT_D.2	JR262_1597_2	SG2	−54.396	−37.384	174.98	ZSM-A20160710	KY272305		x	x
*P. obstaculumsuperavit* sp. nov.	ANT_D.2	JR262_1903_1	SR	−53.597	−41.214	132.83	ZSM-A20160711	KY272303		x	
*P. obstaculumsuperavit* sp. nov.	ANT_D.2	JR262_48_5_2	SG1	−54.284	−36.083	124.08	ZSM-A20160713	KY272298		x	x
*P. obstaculumsuperavit* sp. nov.	ANT_D.2	JR287_124_1	SG1	−53.764	−36.681	151	ZSM-A20160691	KY272295	KY272393	x	x
*P. obstaculumsuperavit* sp. nov.	ANT_D.2	JR287_124_2	SG1	−53.764	−36.681	151	ZSM-A20160692	KY272294	KY272391	x	
*P. obstaculumsuperavit* sp. nov.	ANT_D.2	JR287_152	SG1	−53.758	−36.690	145	ZSM-A20160694	KY272292			x
*P. obstaculumsuperavit* sp. nov.	ANT_D.2	PS77_211_6_1_3	SR	−53.402	−42.668	290.2	ZSM-A20160696	KY272306	KY272395	x	x
*P. latefrontalis*	ANT_F	PS77_226_7_1_2	AP4	−64.915	−60.621	226.2	ZSM-A20160649	KY272334	KY272430	x	x
*P. latefrontalis*	ANT_F	PS77_248_3_2_1	AP5	−65.924	−60.332	433	ZSM-A20160644	KY272337	KY272436	x	x
*P. latefrontalis*	ANT_F	PS77_248_3_2_2	AP5	−65.924	−60.332	433	ZSM-A20160645	KY272336	KY272431	x	
*P. latefrontalis*	ANT_F	PS77_248_3_2_3	AP5	−65.924	−60.332	433	ZSM-A20160646	KY272338	KY272435		x
*P. latefrontalis*	ANT_F	PS77_248_3_2_4	AP5	−65.924	−60.332	433	ZSM-A20160647	KY272332		x	
*P. latefrontalis*	ANT_F	PS77_257_2_2_3	AP4	−64.913	−60.648	152.5	ZSM-A20160650	KY272330	KY272440	x	
*P. latefrontalis*	ANT_F	PS77_257_2_2_5	AP4	−64.913	−60.648	152.5	ZSM-A20160651	KY272329	KY272432	x	
*P. latefrontalis*	ANT_F	PS77_275	WS6	−70.940	−10.489	225.5	ZSM-A20160728	KY272326	KY272439	x	
*P. latefrontalis*	ANT_F	PS77_291_1_2	WS6	−70.842	−10.587	267.5	ZSM-A20160642	KY272333	KY272437	x	
*P. latefrontalis*	ANT_F	PS77_292_2_5	WS6	−70.846	−10.593	243.5	ZSM-A20160729	KY272327	KY272441	x	x
*P. latefrontalis*	ANT_F	PS82_58_1	WS3	−76.322	−29.002	228.5	ZSM-A20160627	KY272328	KY272438	x	
*P. latefrontalis*	ANT_F	ACE2017 1069_2_1_1	BI	−54.425	35.241	327	ZSM-A20190284			x	
*P. latefrontalis*	ANT_F	ACE2017 1069_2_1_2	BI	−54.425	35.241	327	ZSM-A20190285			x	x
*P. latefrontalis*	ANT_F	ACE2017 1069_2_1_3	BI	−54.425	35.241	327	ZSM-A20190286			x	x
*P. hiemalis*	ANT_K	PS82_143_2_2	WS2	−76.967	−32.866	293.7	ZSM-A20160624	KY272325	KY272425	x	
*P. hiemalis*	ANT_K	PS82_244_4	WS5	−72.799	−19.495	739.7	ZSM-A20160640	KY272323		x	x
*P. hiemalis*	ANT_K	PS82_246_2	WS6	−70.928	−10.475	213.5	ZSM-A20160641	KY272324	KY272424	x	
*Pallenopsis* sp. ANT_L	ANT_L	PS82_34_2	WS3	−76.069	−30.160	473	ZSM-A20160628	KY272340	KY272421	x	
*Pallenopsis* sp. ANT_L	ANT_L	PS82_109_2_2	WS2	−77.016	−33.695	435.2	ZSM-A20160622	KY272339	KY272420	x	x
*P. buphtalmus*	ANT_M	PS82_183_1_1	WS1	−74.250	−37.749	833.5	ZSM-A20160638	KY272321	KY272400	x	x
*P. buphtalmus*	ANT_M	PS82_183_1_2	WS1	−74.250	−37.749	833.5	ZSM-A20160639	KY272320	KY272401	x	x
*P. buphtalmus*	ANT_M	PS82_240_2	WS4	−74.660	−28.763	769	ZSM-A20160731	KY272322	KY272402	x	
*P. buphtalmus*	ANT_M	PS96_220_3_1	WS4	−74.657	−26.896	421.8	ZSM-A20190287			x	
*Pallenopsis* sp. ANT_N	ANT_N	PpaE_002_HT25	SG1	−54.016	−37.437	78	ZSM-A20160718	KC794960		x	x
*Pallenopsis* sp. ANT_N	ANT_N	PS77_211_6_1_4	SR	−53.402	−42.668	290.2	ZSM-A20160697	KY272360	KY272458	x	
*P. patagonica*	SUB_1	PS77_208_5_1_1	SP	−56.168	−54.548	292	ZSM-A20160726	KY272289	KY272367	x	x
*P. patagonica*	SUB_1	PS77_208_5_1_4	SP	−56.168	−54.548	292	ZSM-A20160689	KY272288		x	x
*P. patagonica*	SUB_2	ZSMA20111352_HT27	FI1	−51.269	−62.952	171–174	ZSM-A20111352	KF603937/ CFAP037–11		x	x
*P. patagonica*	SUB_2	HF26_254	Pa2	−53.007	−73.923	31	ZSM-A20160456	KY272290	KY272368	x	x
*P. patagonica*	SUB_2	PS77_208_3	SP	−56.152	−54.530	285.5	ZSM-A20160725	KY272291	KY272366	x	x
*P. notiosa*	SUB_3	ZSMA20111008_HT28	Pa3	−50.414	−74.559	15–20	ZSM-A20111008	KF603952/ CFAP026–11	KY272390	x	x
*P. patagonica*	SUB_4	PpaE_004_HT18	FI2	−52.574	−60.084	378	ZSM-A20160719	KC794961	KY272443	x	
*P. patagonica*	SUB_4	PpaE_007_HT15	FI2	−52.574	−60.084	378	ZSM-A20160722	KC794964			x
*P. patagonica*	SUB_4	PS77_208_5_1_2	SP	−56.168	−54.548	292	ZSM-A20160727	KY272356		x	
*P. patagonica*	SUB_4	ZDLT1_889_2	FI1	− 50.252	−61.567	159	ZSM-A20160699	KY272358	KY272446		x
*P. patagonica*	SUB_4	ZDLT1_889_3	FI1	−50.252	−61.567	159	ZSM-A20160700	KY272359	KY272444	x	x
*P. patagonica*	SUB_5	HF26_027	Pa2	−52.600	−73.640	19	ZSM-A20160452	KY272344		x	x
*P. patagonica*	SUB_5	HF26_367	Pa2	−53.357	−73.087	20	ZSM-A20160468	KY272351	KY272447	x	x
*P. patagonica*	SUB_5	HF26_373	Pa2	−53.379	−73.159	14	ZSM-A20160488	KY272347	KY272453		x
*P. patagonica*	SUB_5	HF26_392	Pa2	−53.379	−73.159	17	ZSM-A20160493	KY272345	KY272450		x
*P. patagonica*	SUB_5	KT982315	Pa1	−53.270	−66.386	96		KT982315	KY272456	x	
*P. yepayekae*	Pye.1	HF16_476_2	Pa3	−50.353	−75.283	20	ZSM-A20160580	KY272283		x	
*P. yepayekae*	Pye.1	HF24_213	Pa4	−46.723	−75.255	31.4	ZSM-A20160529	KY272268	KY272372	x	
*P. yepayekae*	Pye.1	HF26_029	Pa2	−52.600	−73.640	15–20	ZSM-A20160450	KY272281		x	x
*P. yepayekae*	Pye.1	HF26_363	Pa2	−53.007	−73.923	20	ZSM-A20160462	KY272284			x
*P. yepayekae*	Pye.1	HF26_378	Pa2	−53.379	−73.159	29	ZSM-A20160498	KY272277	KY272387		x
*P. pilosa*	outgroup	PS96_004_3					ZSM-A20190288			x	

Species names are given if possible. Sampling details (location, latitude, longitude, depth in m), specimen information (ID, voucher number, molecular clade and sequence availability) and analyses applied to each individual (target hybrid enrichment, morphometric analyses) are listed

### Bait enrichment

For genetic analyses, a target hybrid enrichment approach was chosen. For the present analyses we used the bait set designed in Dietz et al. [22] using the workflow described by Mayer et al. [59]. Briefly, the baits were constructed based on an assembly of transcriptomic data of the sea spider *Colossendeis megalonyx* and included a total number of 12,014 baits covering 3682 bait regions from 1607 single-copy EOGs present in all spider genomes. See Dietz et al. [22] for details and bait sequences. Baits were manufactured by Agilent Technologies (Waldbronn, Germany).

Sample preparation was conducted following a slightly modified version of Agilent’s protocol “200 ng DNA sample” for “Agilent’s SureSelect Target Enrichment System”. A detailed written protocol is provided in Additional file 9, Protocol 1. After the enrichment steps, samples were pooled in equimolar ratios for sequencing. Two pools were prepared, containing 32 samples each. Libraries were sent to GATC Biotech GmbH (Konstanz, Germany) for sequencing on an Illumina MiSeq platform using the V2 2 × 250 bp paired-end sequencing kit. 5% PhiX spike-in was added to each run to increase sequencing diversity and hence improve the signal of sequences. Upon delivery, the NGS reads were adapter- and quality-trimmed with fastq-mcf r. 488 [5]. The raw data are available from NCBI Sequence Read Archive (BioProject ID PRJNA544606). We used two complementary approaches to construct data sets, SNP and EOG, from the reads for different purposes. The SNP approach was used to call variants of different sample sets and also include flanking regions. The EOG approach is solely based on orthologous regions and hence is supposed to cover genes only.

### SNP analyses

As there is no reference genome for sea spiders available, a de novo reference based on all raw reads from the samples of the *P. patagonica* species complex was generated with a pipeline of custom Bash shell scripts including quality filtering, sequence editing and assembly. Further information is provided in Additional file 9, Protocol 2. SNPs were called separately for three different data sets: i) all samples belonging to the *P. patagonica* species complex, ii) *P. patagonica* samples belonging to the “Patagonian supergroup”, and iii) *P. patagonica* samples belonging to the “Antarctic supergroup”; see results section for group assignment) to maximize the number of group-specific SNPs (see Additional file 9, Protocol 3 for more information). To analyse the genetic structure, PCAs were conducted using the R-package SNPRelate v. 1.12.2 [85] with default parameters. sNMF-plots were calculated to investigate the number of genetic clusters within the dataset, using the LEA package v. 2.0.0 [31]. A range of K values (number of ancestral populations) in the interval of 1–20 were tested. The number of repetitions was set to 40 with 40,000 iterations and the lowest cross-entropy per K value was determined and plotted to choose the most likely K value. To also analyze the relationships between clusters, a maximum likelihood tree based on the SNP data was obtained with SNPhylo v. 20,140,701 [50].

### Orthology assignment and phylogenetic analyses

For the bait construction, Dietz et al. [22] had searched OrthoDB 9.1 [84] for orthologous single-copy genes present in all four spider (Araneae) genomes. Using Orthograph v. 0.5.14 [67] these genes were aligned on the amino acid level and hidden Markov models (HMMs) were created. With the aid of Orthograph, these HMMs were then reused to mine the transcriptome of *P. patagonica* for the EOGs of interest, as was previously done for *C. megalonyx* [22]. As the baits were originally designed for *Colossendeis*, the *Pallenopsis* genes were aligned with their *Colossendeis* homologs using MAFFT v. 7.305b [46]. Regions that were aligned to the *Colossendeis* bait sequences and which were at least 30 bp in length were extracted. The trimmed Illumina reads were mapped against these regions with the BWA-MEM algorithm in bwa v. 0.7.17 (available from: https://sourceforge.net/projects/bio-bwa/files). Default parameters were used, except that the minimum match length was set to 30 bp. Successfully mapped reads were mapped again against the full coding sequences from the corresponding contigs with bwa as described above. Diploid consensus sequences of the regions matching the reference were generated for each specimen with samtools v. 1.6 [53] and bcftools v. 1.6 (available from: https://github.com/samtools/bcftools). As the consensus sequences were already aligned to the reference sequence, no further alignment was necessary and all sequences were already in the correct reading frame. All gene alignments were then concatenated to one supermatrix of nucleotide sequences, which was used in a maximum likelihood phylogenetic analysis with IQ-TREE v. 1.5.4 [66]. The alignment was partitioned by codon positions and the optimal partitioning scheme was selected with an algorithm implemented in ModelFinder [11, 45] using the Bayesian Information Criterion. A phylogenetic tree search was conducted with IQ-TREE using the selected models, and branch support values were determined from 1000 ultrafast bootstrap replicates.

For rooting the tree, we mined the published transcriptome of *Anoplodactylus insignis* (NCBI accession number SRX2544807) for the genes of interest using Orthograph with the same procedure as described above. *Anoplodactylus insignis* belongs to the Phoxichilidiidae, a family thought to be related to the Pallenopsidae [3, 71]. Amino acid sequences of *A. insignis* were added to the translated genes alignments with MAFFT using the –add option. EOGs for which no *A. insignis* sequence was found and positions present in less than 50% of the taxa were removed. Outlier sequences were excluded with the OLIinSeq program by CM (available upon request) as described in Dietz et al. [22]. After the root of the tree was determined, further analyses were carried out with the nucleotide data sets excluding *A. insignis*.

### Selection tests

Comparative sequence analyses based on stochastic evolutionary models within HyPhy v. 2.3.13 [47] were used to test for selection. The alignment described in the previous section excluding *A. insignis* was used, additionally filtering out all positions present in less than 50% of the samples. All analyses were based on the phylogenetic tree obtained with IQ-TREE (see above), as we expect all genes to have evolved according to the same phylogeny. Furthermore, either the default or settings recommended by the authors of the programs were used. FUBAR [63] and MEME [65] were used to test for selection across sites. Genes with codons under selection (FUBAR: pp. ≥ 0.99; MEME: *p* ≤ 0.01) that were recognised with both methods were used for further branch-site tests, namely, aBSREL [73] and BUSTED [64]. Here, both terminal and internal branches were tested.

### Morphology

Specimens from the different mitochondrial clades of the *P. patagonica* species complex were studied using light microscopy and μCT. For identification, preparation and analyses of individuals, Leica DMRD and Leica DM5000B microscopes were used. Accurate pictures were taken using the Olympus Stylus TG-4 camera (Microscope mode for automatic generation of extended depth of field images). To obtain a 3D reconstruction of one individual per clade without damaging the specimen, a Phoenix Nanotom (GE Sensing & Inspection Technologies, Wunstorf, Germany) cone beam CT scanner was used at voltages of 80 kV to 120 kV and currents of 90 to 140 μA for 53 min. 1440 radiographs were saved and analysed with the integrated software and VGStudio Max v. 2.2.2 (64 bit; Isosurface and Volume Rendering).

Morphometric body measurements were carried out using the digital caliper from MarCal IP67 (Mahr Metrology, Germany). Measurements follow those applied by Dietz et al. [23, 24], with a focus on characters evaluated as useful for species delimitation, and characters that are potentially linked to fitness differences. The latter include i) the proboscis with terminal mouth, which takes up and processes food; ii) the cheliphores, which function as devices to hold the prey/food and moving it to the mouth opening; and iii) the walking legs. When all limbs were present, up to 135 measurements per specimen were taken (Table 4). However, due to damage during trawling, transport, storage or preceding genetic analysis, distal leg articles were often missing and as a result, not all limbs could be measured. Due to the bilateral symmetry of the body, the averaged measurements of the left and right appendages (legs, palps and cheliphores) were used to reduce the amount of missing values. Ovigeral articles, which are appendices specific to sea spiders and used by males to carry fertilised eggs, were excluded from further analyses, to avoid a bias caused by sexual dimorphism.

**Table 4:** List of characters measured for morphometric analyses

Abbreviation	Description
trunk L	total length of trunk
ceph. segment	length of cephalic segment
trunk W1	diameter of lateral process of 1st trunk segment
trunk W12	width of trunk between 1st and 2nd lateral processes
trunk W2	diameter of lateral process of 2nd trunk segment
trunk W23	width of trunk between 2nd and 3rd lateral processes
trunk W3	diameter of lateral process of 3rd trunk segment
trunk W34	width of trunk between 3rd and 4th lateral processes
trunk W4	diameter of lateral process of 4th trunk segment
trunk H	height of trunk
abdomen L	length of abdomen
abdomen W	width of abdomen
ocular tubercle H	height of ocular tubercle
ocular tubercle W	width of ocular tubercle
eye H	height of anterior eye
forehead H	distance between eyes and apex of ocular tubercle
eyes distance	distance between eyes
proboscis L	proboscis length
proboscis basis	diameter of proboscis at proximal basis
proboscis thickest	diameter of proboscis at thickest part of proboscis
proboscis thick2tip	distance between tip of proboscis and thickest part
proboscis thinnest	diameter of proboscis at thinnest part of proboscis
proboscis thin2tip	distance between tip of proboscis and thinnest part
l/r palp	length of palp bulb
l/r cheliphore 1–3	length of first 3 cheliphore articles; left and right
l/r cheliphore 4	ultimate cheliphore article (moveable finger)
l/r oviger 1–10	length of all 10 ovigeral articles; left and right
l/r WL1–4 coxa1	length of 1st coxa for all 4 pairs of walking leg; left and right
l/r WL1–4 coxa2	length of 2nd coxa for all 4 pairs of walking leg; left and right
l/r WL1–4 coxa3	length of 3rd coxa for all 4 pairs of walking leg; left and right
l/r WL1–4 femur	length of femur for all 4 pairs of walking leg; left and right
l/r WL1–4 tibia1	length of 1st tibia for all 4 pairs of walking leg; left and right
l/r WL1–4 tibia2	length of 2nd tibia for all 4 pairs of walking leg; left and right
l/r WL1–4 tarsus	length of the tarsus for all 4 pairs of walking leg; left and right
l/r WL1–4 propodus	length of the propodus for all 4 pairs of walking leg; left and right
l/r WL1–4 claw	length of the claw for all 4 pairs of walking leg; left and right
l/r WL1–4 aux. claw	length of auxiliary claw for all 4 pairs of walking leg; left and right

Description of how characters were measured and abbreviations for all as used in Table 3 and Additional file 4

For analyses of morphometric measurements Past v. 3.18 [36] was used. First, measurements were tested for normality distribution using the Anderson-Darling, Shapiro-Wil, and Jarque-Bera tests. PCAs were performed to visualise the clustering of specimens regardless of predefined clades and missing values were handled as “iterative imputation” as recommended in Past Manual [35]. In addition, row-wise bootstrapping was carried out using *N* = 1000. Also, LDAs was performed and confusion matrices calculated.

To cope with the missing data points and to limit the analysis to clades with a minimal representation, we pre-filtered the data set to leave only clades with a minimum of three individuals and characters with not more than 10% missing values. Remaining missing values were imputed using Predictive Mean Matching. Analyses were performed using both absolute values of measurements, and relative lengths of measurements expressed as proportion of the trunk to reduce biases caused by different absolute sizes.

Since the number of the characters was large with respect to the number of the individuals, a selection of characters for LDA was performed to avoid model overfitting. The heuristic search for the optimal sets of characters was carried out by iteratively using the stepclass function from the R package klaR v. 0.6–14 with forward-backward selection direction, cross-validation correctness rate as the optimality criterion (taking ten folds) and 5 as the maximum number of characters in a set. The search was organised by picking each one of the characters as starting variable and repeating the procedure ten times. The performance of the character sets was recorded and the best set was used for a final LDA.

Finally, nonparametric unifactorial Kruskal-Wallis H in combination with Dunn’s post hoc test (Bonferroni-corrected) were used to test for significant differences between geographic (sub-Antarctic vs Antarctic) and genetic groupings as well as sexes.

### Combining morphological and genetic data

To test whether there are greater morphological differences for taxa living in sympatry in contrast to those living in allopatry, which can be expected in case of adaptive divergence, pairwise morphological distances were calculated in Past. Subsequently, those were compared with uncorrected pairwise genetic distances calculated in MEGA7 [49]. To be able to calculate genetic distances between all morphologically analysed specimens, COI sequences were used as enrichment data were not available for all specimens. In addition, genetic distances between specimens that were also used for target hybrid enrichment were calculated using the EOG sequence alignment. Linear regression between values and significant differences between ranges of genetic distances were again evaluated in Past.

## Additional files

Additional file 1: Phylogenetic EOG tree of the *Pallenopsis patagonica *species complex. Maximum-Likelihood tree based on concatenated EOG sequences of all samples using *P. pilosa* and transcriptomic data of *Anoplodactylus insignis* as outgroup. Bootstrap values are given next to the respective branches. (PDF 296 kb)

Additional file 2: Phylogenetic SNP tree of the *Pallenopsis patagonica *species complex. Maximum-Likelihood tree based on aligned SNP data of all Pallenopsis samples. Bootstrap values are given next to the respective branches. (PDF 330 kb)

Additional file 3: Cross-entropy estimates of genomic sNMF analysis of the *Pallenopsis patagonica* species complex. Figure showing cross-entropy estimates of genomic sNMF analysis of the *Pallenopsis patagonica* species complex for 1 to 20 ancestral populations (K value). (PDF 263 kb)

Additional file 4: Morphological measurements of the *Pallenopsis patagonica* species complex. Table including all measured characters of all individuals used for morphometric analyses. Detailed information about ID, voucher number, sex as well as species and mitochondrial clade assignment is given. Missing values are substituted with a question mark (?). (XLS 158 kb)

Additional file 5: PCA from morphological data of the *Pallenopsis patagonica* species complex. PCA plots based on morphological measurements. All mitochondrial clades are indicated by different symbols. Symbols of samples from Patagonian (SUB) have no filling, in contrast to the filled symbols of Antarctica (ANT). Male specimens have a blue, females a red outline. (PDF 569 kb)

Additional file 6: Matrices of PCA plots based on reduced morphometric data sets of the *Pallenopsis patagonica* species complex. All combinations of all five axes (PCs) are represented for data sets including A) absolute and B) relative values. Each color represents a different clade (see legend). (PDF 677 kb)

Additional file 7: Morphological distances against genomic distances. Figure showing morphological distances plotted against genomic distances (based on target hybrid enrichment data) between individuals of the *Pallenopsis patagonica* species complex. Red: intraspecific distances (the rightmost red squares represent intraclade distances of SUB_2); grey: interspecific distances. Linear regression line is given (r = 0.51, *p* <  0.0001). (PDF 248 kb)

Additional file 8: Summary of information used for species delimitation of the *Pallenopsis patagonica* species complex. Table including all information used for species delimitation of the *Pallenopsis patagonica* species complex. For species delimitation, morphological and genetic analyses were considered. Previously published results are also included. Black filling indicates missing data. (XLS 78 kb) (XLS 78 kb)

Additional file 9: Detailed protocols of methods used. Detailed written protocols for bait enrichment, de novo reference assembly and SNP calling as performed for analyses within the study. (PDF 177 kb)
