# Supplementary material for: Combining morphological and genomic evidence to resolve species diversity and study speciation processes of the Pallenopsis patagonica (Pycnogonida) species complex
Source: Front Zool. 2019 Sep 6;16:36. doi: 10.1186/s12983-019-0316-y (PMC6728986; doi:10.1186/s12983-019-0316-y)
Supplement: Supplementary file 1 — Phylogenetic EOG tree of the Pallenopsis patagonica species complex. Maximum-Likelihood tree based on concatenated EOG sequences of all samples using P. pilosa and transcriptomic data of Anoplodactylus insignis as outgroup. Bootstrap values are given next to the respective branches. (PDF 296 kb) [file 12983_2019_316_MOESM1_ESM.pdf]

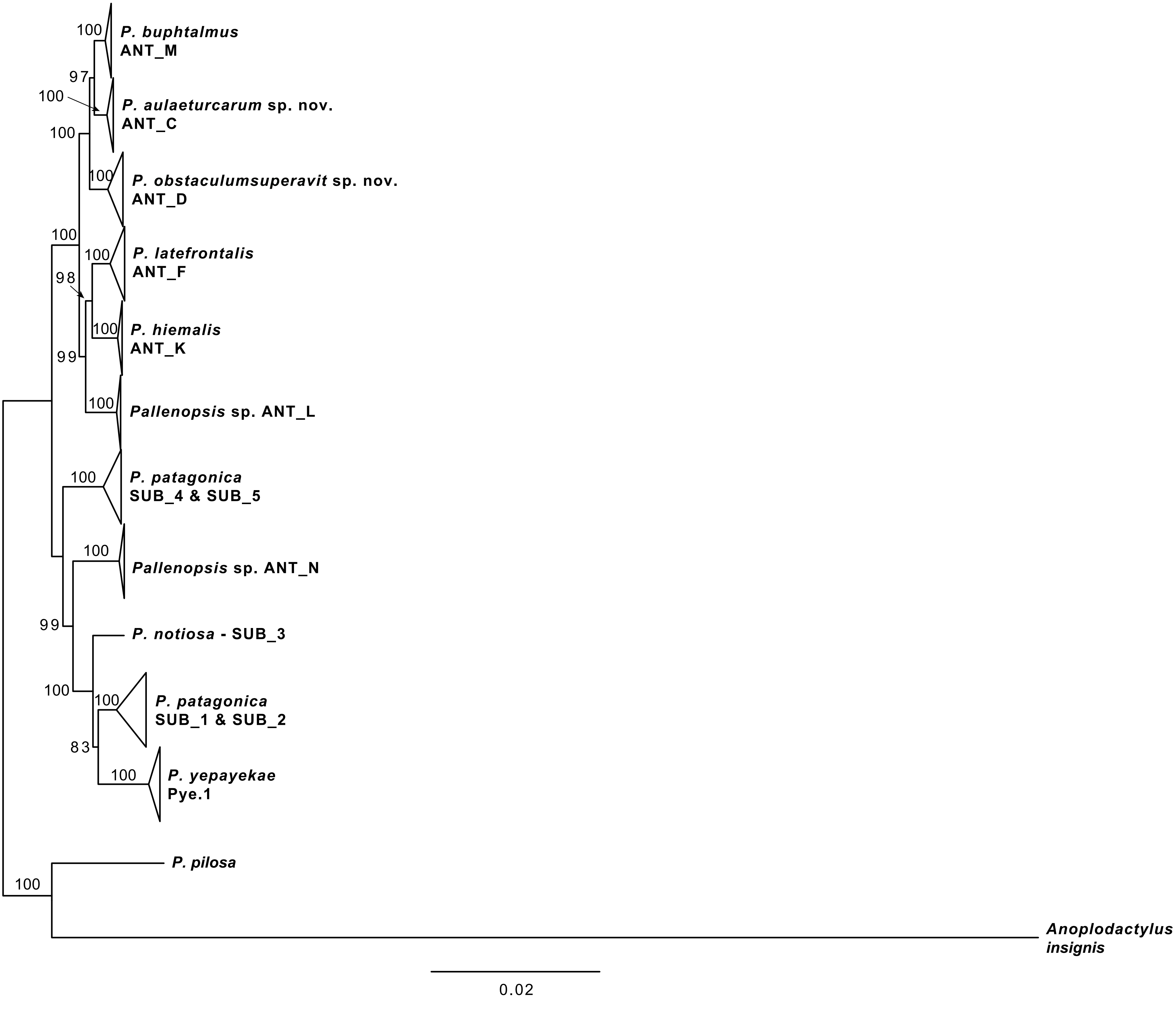

Additional file 1: Phylogenetic EOG tree of the *Pallenopsis patagonica* species complex. Maximum-Likelihood tree based on concatenated EOG sequences of all samples using *P. pilosa* and transcriptomic data of *Anoplodactylus insignis* as outgroup. Bootstrap values are given next to the respective branches.
