# Supplementary material for: Combining morphological and genomic evidence to resolve species diversity and study speciation processes of the Pallenopsis patagonica (Pycnogonida) species complex
Source: Front Zool. 2019 Sep 6;16:36. doi: 10.1186/s12983-019-0316-y (PMC6728986; doi:10.1186/s12983-019-0316-y)
Supplement: Supplementary file 2 — Phylogenetic SNP tree of the Pallenopsis patagonica species complex. Maximum-Likelihood tree based on aligned SNP data of all Pallenopsis samples. Bootstrap values are given next to the respective branches. (PDF 330 kb) [file 12983_2019_316_MOESM2_ESM.pdf]

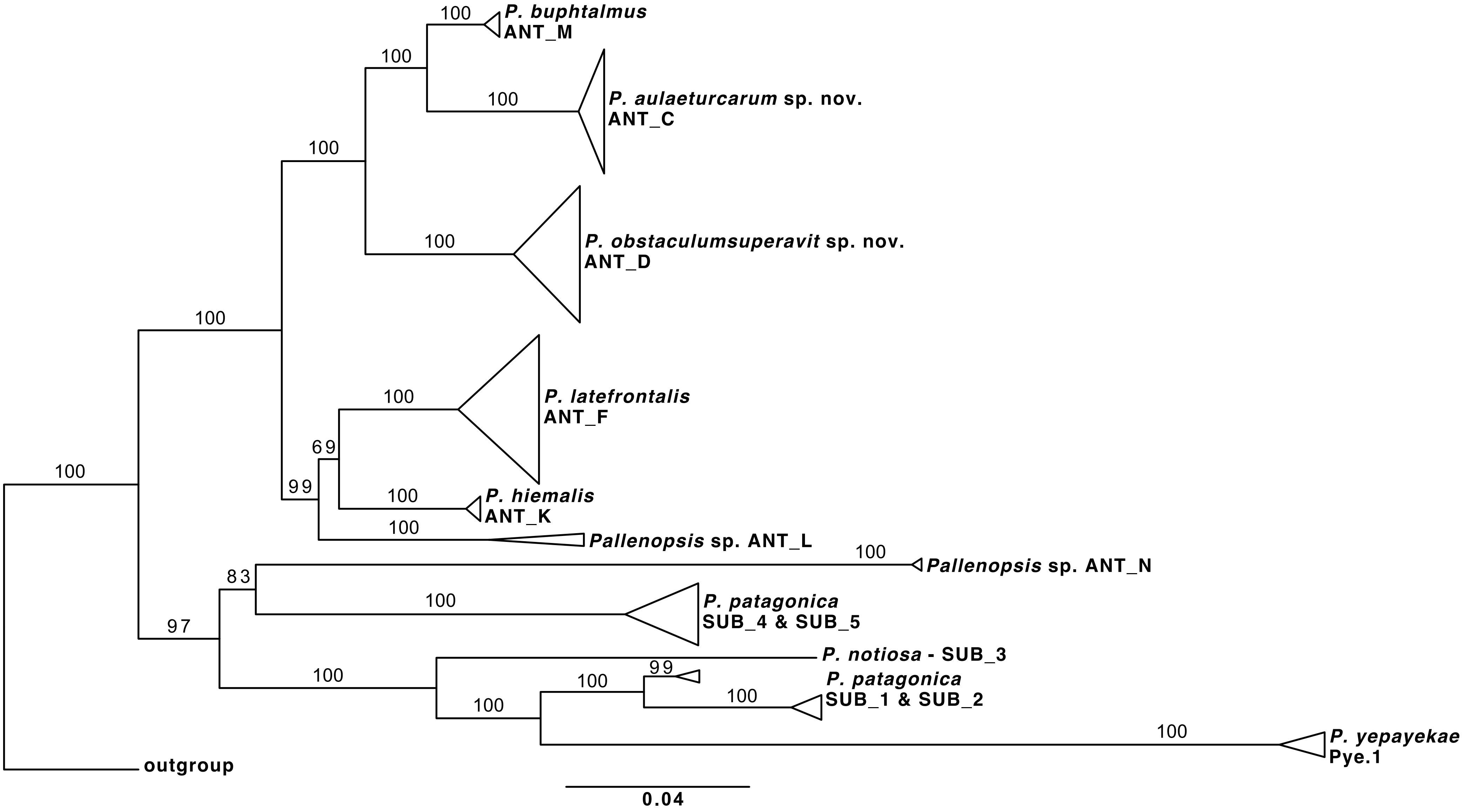

Additional file 2: Phylogenetic SNP tree of the *Pallenopsis patagonica* species complex. Maximum-Likelihood tree based on aligned SNP data of all *Pallenopsis* samples. Bootstrap values are given next to the respective branches.
