# Supplementary material for: Combining morphological and genomic evidence to resolve species diversity and study speciation processes of the Pallenopsis patagonica (Pycnogonida) species complex
Source: Front Zool. 2019 Sep 6;16:36. doi: 10.1186/s12983-019-0316-y (PMC6728986; doi:10.1186/s12983-019-0316-y)
Supplement: Supplementary file 3 — Cross-entropy estimates of genomic sNMF analysis of the Pallenopsis patagonica species complex. Figure showing cross-entropy estimates of genomic sNMF analysis of the Pallenopsis patagonica species complex for 1 to 20 ancestral populations (K value). (PDF 263 kb) [file 12983_2019_316_MOESM3_ESM.pdf]

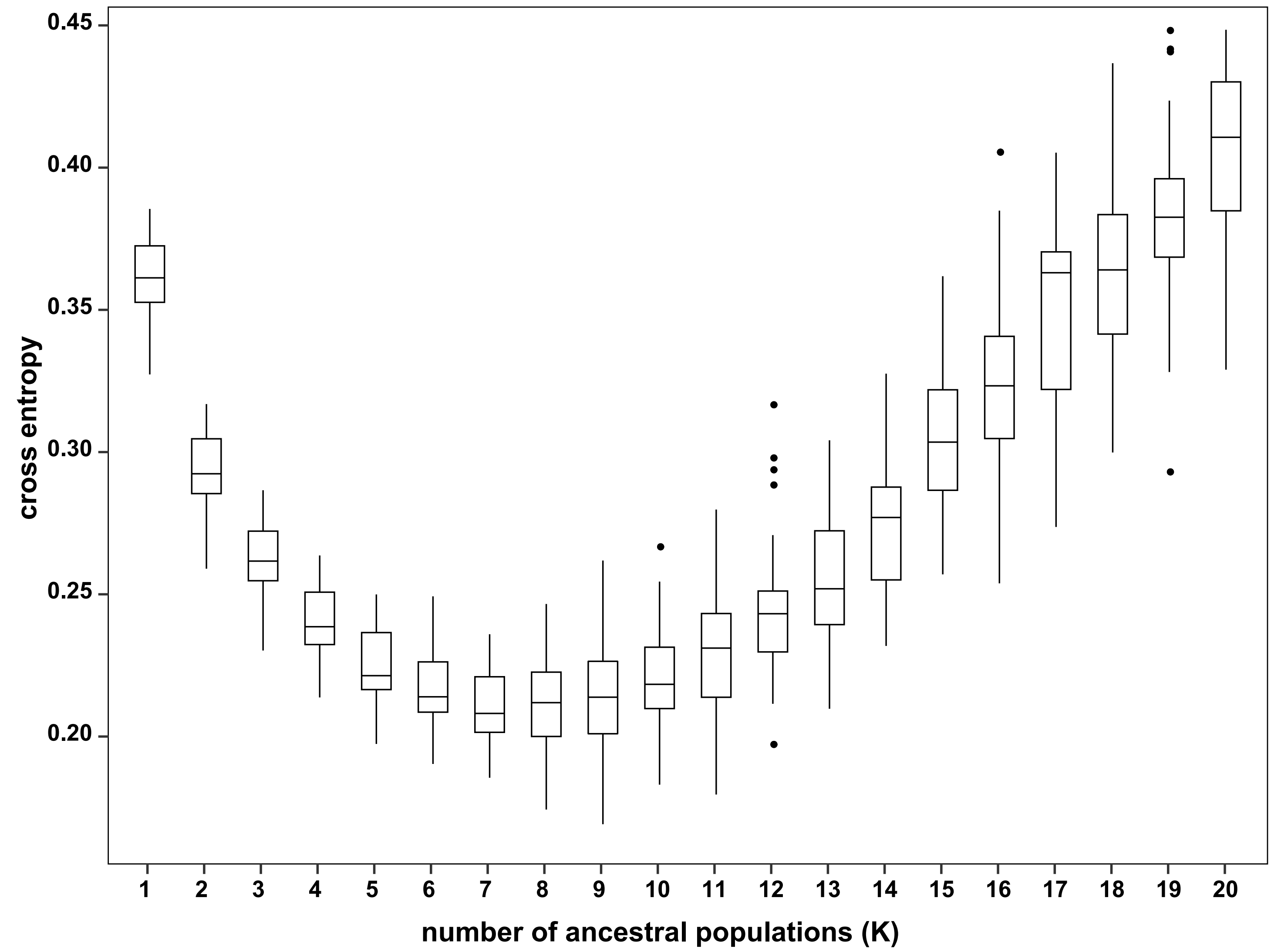

Additional file 3: Cross-entropy estimates of genomic sNMF analysis of the *Pallenopsis patagonica* species complex for 1 to 20 ancestral populations (K value).
