# Supplementary material for: Combining morphological and genomic evidence to resolve species diversity and study speciation processes of the Pallenopsis patagonica (Pycnogonida) species complex
Source: Front Zool. 2019 Sep 6;16:36. doi: 10.1186/s12983-019-0316-y (PMC6728986; doi:10.1186/s12983-019-0316-y)
Supplement: Supplementary file 5 — PCA from morphological data of the Pallenopsis patagonica species complex. PCA plots based on morphological measurements. All mitochondrial clades are indicated by different symbols. Symbols of samples from Patagonian (SUB) have no filling, in contrast to the filled symbols of Antarctica (ANT). Male specimens have a blue, females a red outline. (PDF 569 kb) [file 12983_2019_316_MOESM5_ESM.pdf]

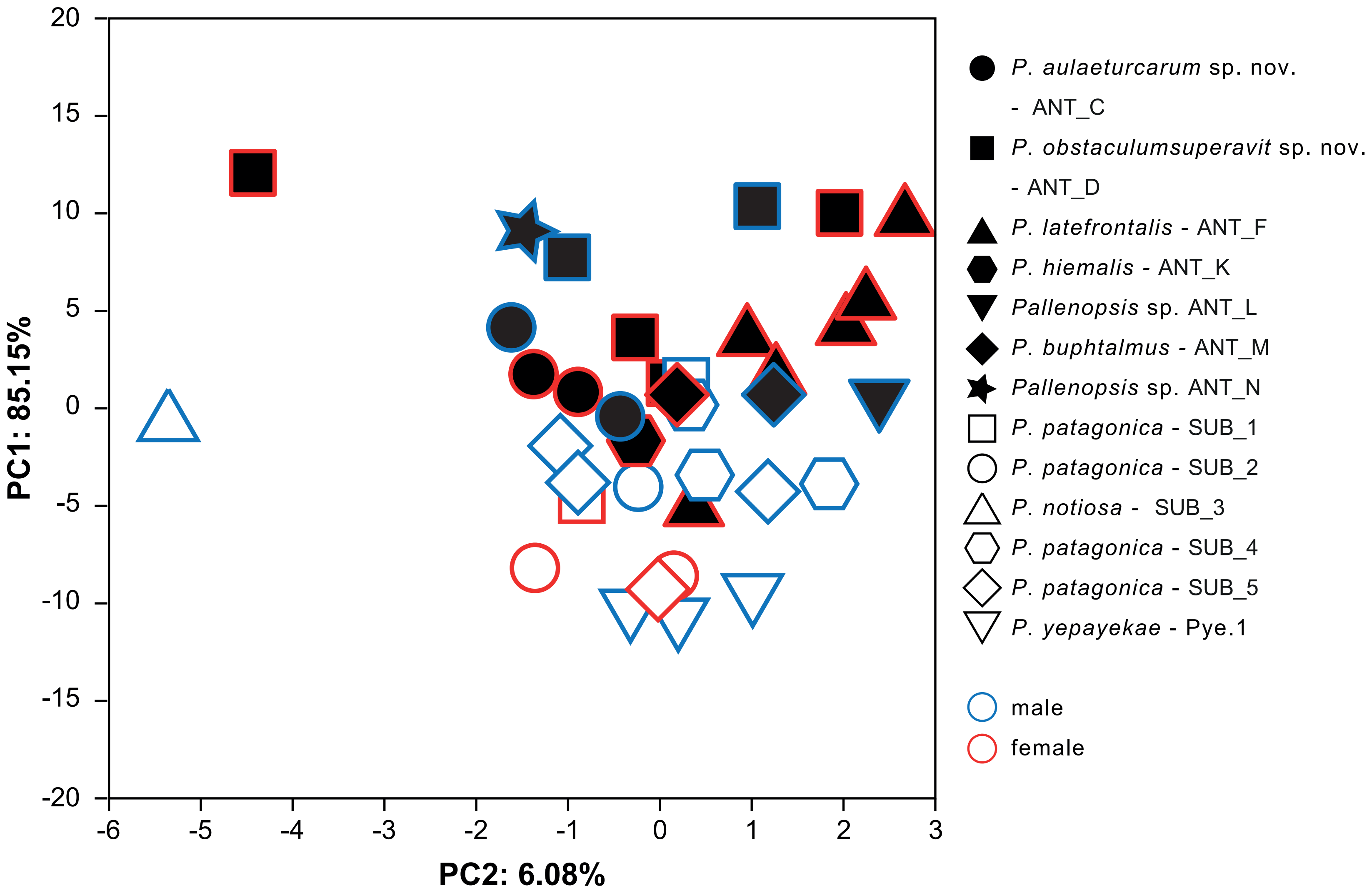

Additional file 5: PCA from morphological data of the *Pallenopsis patagonica* species complex.
PCA plots based on morphological measurements. All mitochondrial clades are indicated by different symbols. Symbols of samples from Patagonian (SUB) have no filling, in contrast to the filled symbols of Antarctica (ANT). Male specimens have a blue, female a red outline.
