# Supplementary material for: Combining morphological and genomic evidence to resolve species diversity and study speciation processes of the Pallenopsis patagonica (Pycnogonida) species complex
Source: Front Zool. 2019 Sep 6;16:36. doi: 10.1186/s12983-019-0316-y (PMC6728986; doi:10.1186/s12983-019-0316-y)
Supplement: Supplementary file 6 — Matrices of PCA plots based on reduced morphometric data sets of the Pallenopsis patagonica species complex. All combinations of all five axes (PCs) are represented for data sets including A) absolute and B) relative values. Each color represents a different clade (see legend). (PDF 677 kb) [file 12983_2019_316_MOESM6_ESM.pdf]

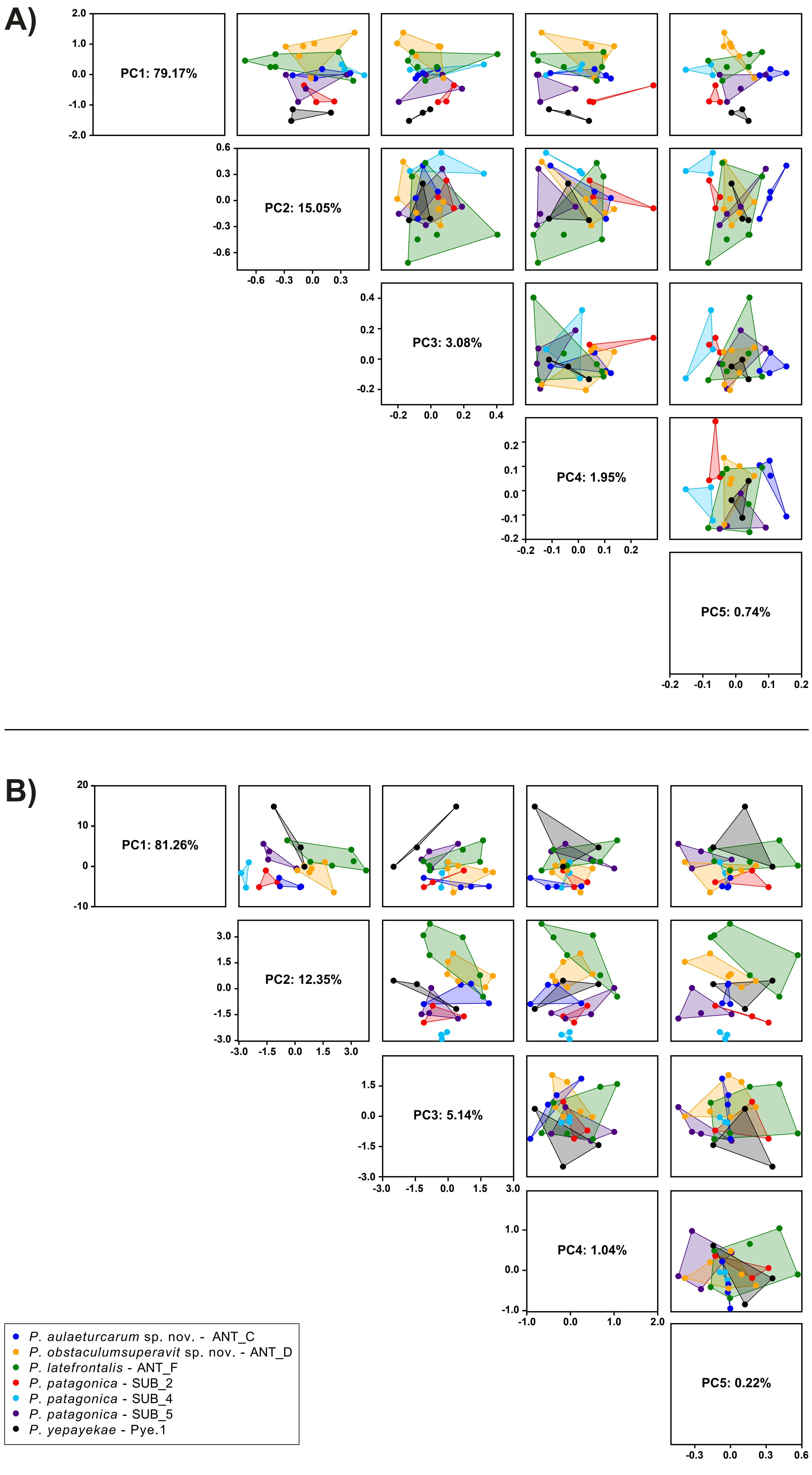

Additional file 6: Matrices of PCA plots based on reduced morphometric data sets of the *Pallenopsis patagonica* species complex. All combinations of all five axes (PCs) are represented for data sets including A) absolute and B) relative values. Each colour represents a different clade (see legend).
