# Supplementary material for: Combining morphological and genomic evidence to resolve species diversity and study speciation processes of the Pallenopsis patagonica (Pycnogonida) species complex
Source: Front Zool. 2019 Sep 6;16:36. doi: 10.1186/s12983-019-0316-y (PMC6728986; doi:10.1186/s12983-019-0316-y)
Supplement: Supplementary file 7 — Morphological distances against genomic distances. Figure showing morphological distances plotted against genomic distances (based on target hybrid enrichment data) between individuals of the Pallenopsis patagonica species complex. Red: intraspecific distances (the rightmost red squares represent intraclade distances of SUB_2); grey: interspecific distances. Linear regression line is given (r = 0.51, p < 0.0001). (PDF 248 kb) [file 12983_2019_316_MOESM7_ESM.pdf]

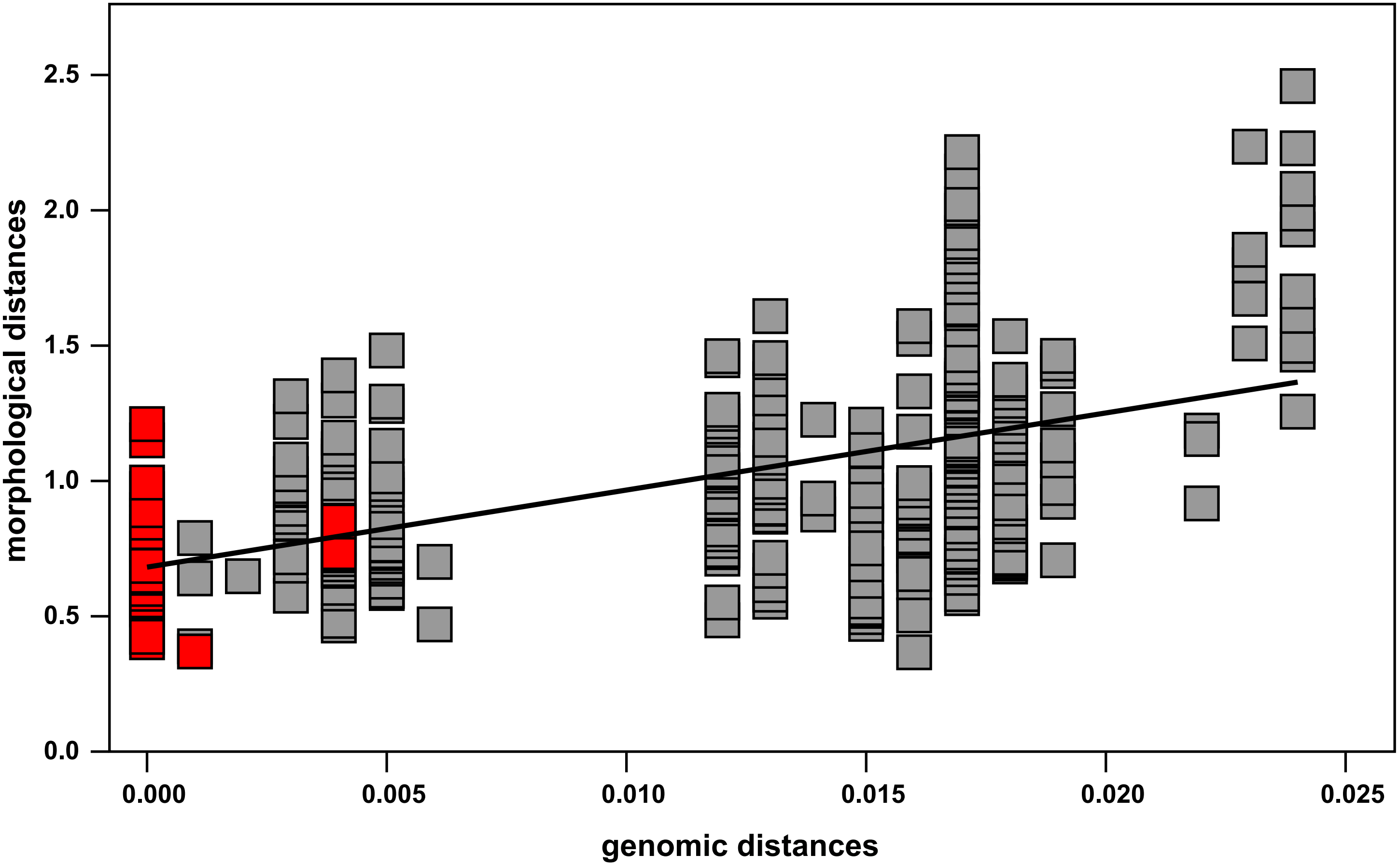

Additional file 7: Morphological distances against genomic distances. Morphological distances plotted against genomic distances (based on target hybrid enrichment data) between individuals of the *Pallenopsis patagonica* species complex. Red: intraspecific distances (the rightmost red squares represent intraclade distances of SUB_2); grey: interspecific distances. Linear regression line is given (r=0.51, p<0.0001).
