# Supplementary material for: Combining morphological and genomic evidence to resolve species diversity and study speciation processes of the Pallenopsis patagonica (Pycnogonida) species complex
Source: Front Zool. 2019 Sep 6;16:36. doi: 10.1186/s12983-019-0316-y (PMC6728986; doi:10.1186/s12983-019-0316-y)
Supplement: Supplementary file 9 — Detailed protocols of methods used. Detailed written protocols for bait enrichment, de novo reference assembly and SNP calling as performed for analyses within the study. (PDF 177 kb) [file 12983_2019_316_MOESM9_ESM.pdf]

Combining morphological and genomic evidence to resolve species diversity and study speciation processes of the *Pallenopsis patagonica* (Pycnogonida) species complex

Jana S. Dömel, Till-Hendrik Macher, Lars Dietz, Sabrina Duncan, Christoph Mayer, Andrey Rozenberg, Katherine Wolcott, Florian Leese, Roland R. Melzer

Additional file 9

Protocol 1: Bait enrichment

For genetic analyses, a target hybrid enrichment approach was chosen and a bait set especially designed for sea spiders was used, but see Dietz et al. (2019) for further details about bait design. The total number of baits was 12,014 in 3,682 bait regions. Baits were produced by Agilent Technologies (Waldbronn, Germany).

Sample preparation was conducted following a slightly modified version of Agilent's protocol "200 ng DNA sample" for "Agilent's SureSelect Target Enrichment System". 100 ng per 10 µl were used for DNA fragmentation in the Bioruptor Pico Sonicaton System (Diagenode SA). One cycle consisted of 30 seconds of ultrasound shearing followed by a 30 second pause (cooling). The optimal number of shearing cycles was determined by the proportion of fragments smaller than 500 bp as determined with Fragment Analyzer using the High Sensitivity NGS Kit (DNF-474-33, Advanced Analytical Technologies). A percentage of short fragments greater than 95% was generally regarded as optimal, but a few difficult samples were accepted with 85% for further preparation. Afterwards, samples were topped off with HPLC water to a total of 10 µl, then stored at 4°C until further preparation. Unless otherwise indicated, the following steps only used half the volumes mentioned in the original protocol. Reaction products for all steps involved in the hybrid enrichment were purified using magnetic beads (Agentcourt AMPure XP Kit, Beckman Coulter Genomics). To enrich for fragments smaller than 500 bp a volume of beads equal to the reaction volume was added to the reaction itself. Steps prior to hybridization included end repairing, adenylation of the 3' end, and adaptor ligation. DNA concentration was measured using the High Sensitivity Kit for Qubit (Thermo Fisher Scientific). Depending on the concentration, amplification of adaptor-ligated samples was performed with 10 to 20 cycles of PCR. A minimum of 375 ng per sample was required for the hybridization step. Samples were dried overnight in a thermal block at 40°C and eluted with HPLC water to a concentration of 220 ng/µl. 1.7 µl was then used for hybridization, performed at 60°C for 24 hours. In contrast to the original protocol, targeted sequences were

caught using 15 µl streptavidin-coated magnetic beads (Dynabeads MyObe Streptavidin C1, Thermo Fisher Scientific). Post-capture PCR with 18 cycles was performed to amplify captured and indexed fragments of each sample individually. Concentrations were measured with Qubit's dsDNA BR Array Kit (Thermo Fisher Scientific). Afterwards, samples were pooled in equal quantities for sequencing. Each pool contained 32 samples (1st library 12.6 ng/µl and 2nd library 21.8 ng/µl). Libraries were sent to GATC Biotech GmbH (Konstanz, Germany) for sequencing on an Illumina MiSeq platform using the V2 2x250 bp paired-end sequencing kit. 5% PhiX spike-in was added in each run.

Protocol 2: *De novo* reference

Since no sea spider genome is currently available, a *de novo* reference had to be produced to be able to map the enrichment data and align sequences of different individuals. The final *de novo* reference was based on the assembly of 63 complex paired-end read samples of the *P. patagonica* species. All pairs of NGS raw read files were initially quality checked using FastQC v. 0.11.5 (Andrews et al. 2010) and then filtered and trimmed with cutadapt v. 1.14 (Martin et al. 2011) (minimum length = 35; Error-rate = 0.1; quality cutoff = 15; overlap minimum length = 3). The trimmed paired reads were assembled with Trinity v. 2.5.1 (Grabherr et al. 2011), using a minimum coverage of 10 and the standard kmer length of 25. As Trinity is an RNA assembler, the output contains isoforms of the same contig. A maximum of two isoforms per contig were allowed to account for potentially heterozygous loci, while contigs with three or more isoforms were removed from the data set to avoid chimeras and paralogous genes. Two isoforms in a pair were aligned using the water program from EMBOSS v. 6.5.7.0 (Li et al. 2015). Consensus sequences were called with consambig from EMBOSS and added to the final *de novo* reference data set alongside single-isoform contigs. The final assembly for the *de novo* reference.

Protocol 3: SNP calling

Trimmed raw reads of all samples were mapped to the reference assembly using BWA mem (Li 2013). Resulting alignment files were subsequently processed with samtools v. 1.6 (Li et al. 2009, Li 2011), by adding read groups, to uniquely identify each read, and marking duplicates to reduce redundancy on the level of individual libraries. Variant calling was conducted with HaplotypeCaller from the GATK v. 4.0.3.0 package (McKenna et al. 2010). Variant calling was performed for three different sample sets. For further analysis, SNPs were extracted using GATK SelectVariants and filtered with VariantFiltration according to the GATK best practices workflow (DePristo et al. 2011) by applying the following thresholds: QualByDepth < 2.0,

FisherStrand > 60.0, RMSMappingQuality < 40.0, StrandOddsRatio > 3.0, MappingQualityRankSum < -12.5 and ReadPosRankSumTest < -8.0. In addition to the 0/0 homozygous and 0/1 heterozygous genotypes filtered by VariantFiltration, the resulting variant files were filtered to include 0/0 homozygous genotypes with VCFFilterJS r. f4c7a81 (Lindenbaum et al. 2018). VCFtools v. 0.1.13 (Danecek et al. 2011) was used to retain only bi-allelic sites with a maximum missing rate of 20% across individuals within a data set. Furthermore, additional vcf files with thinned sites, i.e. one SNP per contig only, were produced. Invariant sites with missing samples, which were neither considered by VCFFilterJS nor by GATK, were filtered with custom script. The vcf files were used as a basis for all subsequent analyses.

References:

Andrews S. FastQC: a quality control tool for high throughput sequence data. 2010.

Danecek P, Auton A, Abecasis G, Albers CA, Banks, E, DePristo MA, et al. The variant call format and VCFtools. Bioinformatics. 2011:27:2156-2158; doi:10.1093/bioinformatics/btr330.

DePristo MA, Banks E, Poplin R, Garimella KV, Maguire JR, Hartl C, et al. A framework for variation discovery and genotyping using next-generation DNA sequencing data. Nature Genetics. 2011;43:491-498; doi:10.1038/ng.806.

Dietz L, Dömel JS, Leese F, Mahon AR, Mayer C. Phylogenomics of the longitarsal Colossendeidae: the evolutionary history of an Antarctic sea spider radiation. Mol Phylogenet Evol. 2019;136:206-214.

Grabherr MG, Haas BJ, Yassour M, Levin JZ, Thompson DA, Amit I, et al. Full-length transcriptome assembly from RNA-Seq data without a reference genome. Nat Biotechnol. 2011;29:644-652; doi: 10.1038/nbt.1883.

Li H. A statistical framework for SNP calling, mutation discovery, association mapping and population genetical parameter estimation from sequencing data. Bioinformatics. 2011;27:2987-2993; doi:10.1093/bioinformatics/btr509.

Li H. Aligning sequence reads, clone sequences and assembly contigs with BWA-MEM. arXiv:1303.3997 [q-bio]. 2013.

Li H, Handsaker B, Wysoker A, Fennell T, Ruan J, Homer N. The Sequence Alignment/Map format and SAMtools. Bioinformatics. 2009;25:2078-2079; doi:10.1093/bioinformatics/btp352.

Li W, Cowley A, Uludag M, Gur T, McWilliam H, Squizzato S. et al. The EMBL-EBI bioinformatics web and programmatic tools framework. Nucleic Acids Res. 2015;43:W580–W584; doi: 10.1093/nar/gkv279.

Lindenbaum P, Redon R, Hancock J. Bioalcidae, samjs and vcffilterjs: object-oriented formatters and filters for bioinformatics files. Bioinformatics. 2018;34:1224-1225; doi:10.1093/bioinformatics/btx734.

Martin M. Cutadapt removes adapter sequences from high-throughput sequencing reads. EMBnet journal. 2011;17:10-12.
